# Supplementary material for: Variation in temperature of peak trait performance constrains adaptation of arthropod populations to climatic warming
Source: Nat Ecol Evol. 2024 Jan 25;8(3):500–10. doi: 10.1038/s41559-023-02301-8 (PMC10927549; doi:10.1038/s41559-023-02301-8)
Supplement: Supplementary file 1 — Supplementary Results including Figs. 1–16 and Table 15. [file 41559_2023_2301_MOESM1_ESM.pdf]

nature ecology & evolution

Article https://doi.org/10.1038/s41559-023-02301-8

Variation in temperature of peak trait performance constrains adaptation of arthropod populations to climatic warming

In the format provided by the authors and unedited

Contents

1 Supplementary Results **2**
1.1 Trait-level “hotter-is-better” patterns . . . 2
1.2 Correlation of thermal fitness with peak trait performance . . . 4
1.3 Evidence of trait-level thermal adaptation . . . 5
1.4 Distributions of trait-level thermal sensitivities . . . 8
1.5 Trait-level thermal performance curves . . . 9
1.6 Species-level temperature dependencies of r_m . . . 13
1.7 Sensitivity of the results to the parameterisation of fecundity loss rate (κ) . . . 14
1.7.1 Effect on the trait sensitivity results . . . 15
1.7.2 The selection gradients revisited . . . 15
1.8 Macroevolutionary patterns and phylogenetic constraints . . . 17

1 Supplementary Results

1.1 Trait-level "hotter-is-better" patterns

Across diverse levels of biological organisation, many biological rates (e.g., development, population growth) are expected to scale to the negative quarter-power with mass-specific metabolic rate ($M^{-0.25}$) but such scaling may not exist in arthropods [1]. Therefore, prior to testing for trait-level hotter-is-better patterns, trait B_{pk}s were temperature- and body mass-corrected to account for how optimal thermal fitness (r_{opt}) emerges from the TPCs of its underlying traits which in turn depend on scaling relationships between body size and metabolic rate in individual organisms [2]. Specifically, to obtain the exponents for these relationships, we fitted OLS models in log-log scale (i.e., $\log(B_{pk}) \sim \log(M) + kT$; where M is fresh wet mass in milligrams, k is the Boltzmann constant (Main text Table 1) and T is the trait value at 273.15°K (0°C)) to the B_{pk} estimates (Main text Fig. 5C and Supplementary Figs. 1 & 2) and the body mass data (Appendix 1). We also corrected r_{opt} to account for size scaling in its underlying traits (Main text Fig. 5B and Supplementary Fig. 2). If fresh mass for a particular species was not provided in the original study, we used mass estimates from other studies on that or a closely related species.

Our theory assumes that a "hotter-is-better" pattern exists in the underlying traits. In particular, given its dominant effect, α should definitely exhibit this pattern. While data on this within-species are not currently available, we tested this assumption with data across species (Main text Fig. 5B; SM Fig. 1). We observe a significant "hotter-is-better" pattern for development time (α; Main text Fig. 5B) and maximum fecundity (b_{max}) (SM Fig. 1A and D), whereas the slopes for z_J and z are non significantly positive and negative, respectively. *Anoplophora glabripennis* was excluded from this analysis because it's r_{opt} was extremely low (r_{opt}=0.01). With this species included, the slope for seen in Main text Fig. 5C becomes 0.09 ± 0.08 (95% CI), R^2=0.21 and p=0.03.

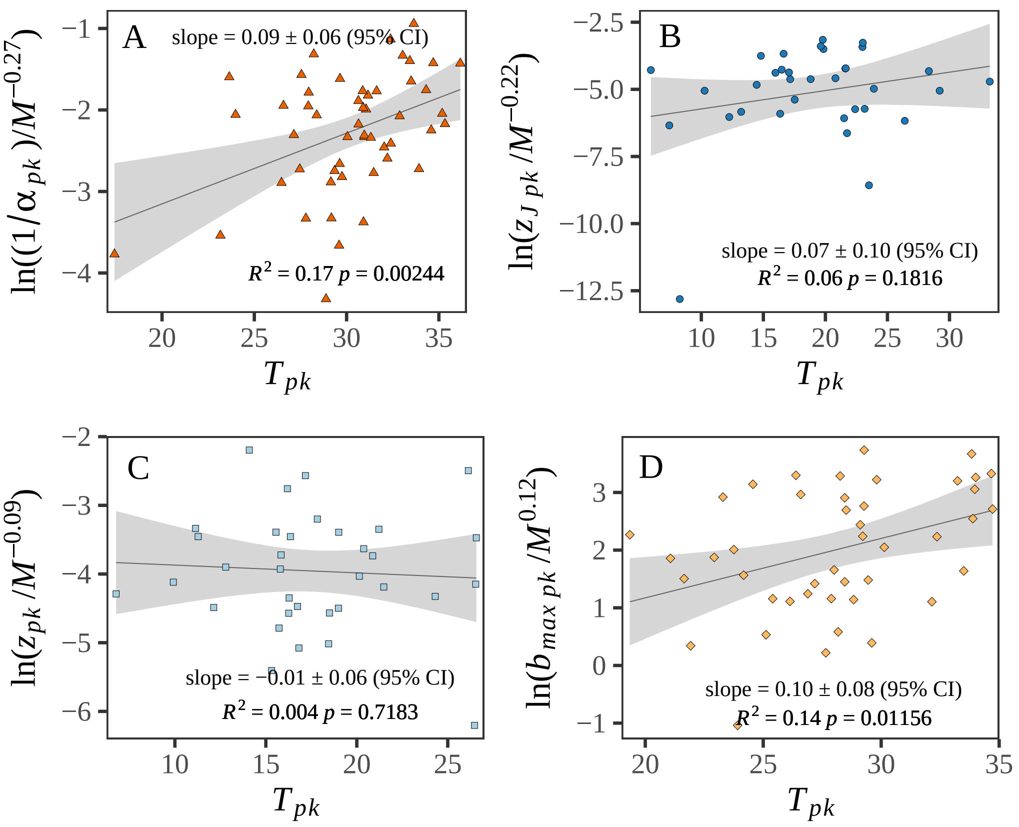

Supplementary Figure 1: Test of the “hotter-is-better” pattern across arthropod taxa. The “hotter-is-better” pattern is significant in the body size-corrected (wet mass, mg) $1/\alpha$ (A; n=51 independent species) and b_{max} (D; n=44 independent species) data, but at best weak for z_J (B; n=34 independent species) and z (C; n=34 independent species), suggesting relatively greater biochemical adaptation to overcome thermodynamic constraints in these two traits [3, 4] , insufficient data (note the narrow range of temperatures on x-axis for b_{max} in particular) [5], or both. The lines are OLS regression (with 95% prediction bounds) fitted to the species' log-transformed median B_{pk}s (symbols) of the trait plotted against their respective median T_{pk}s.

1.2 Correlation of thermal fitness with peak trait performance

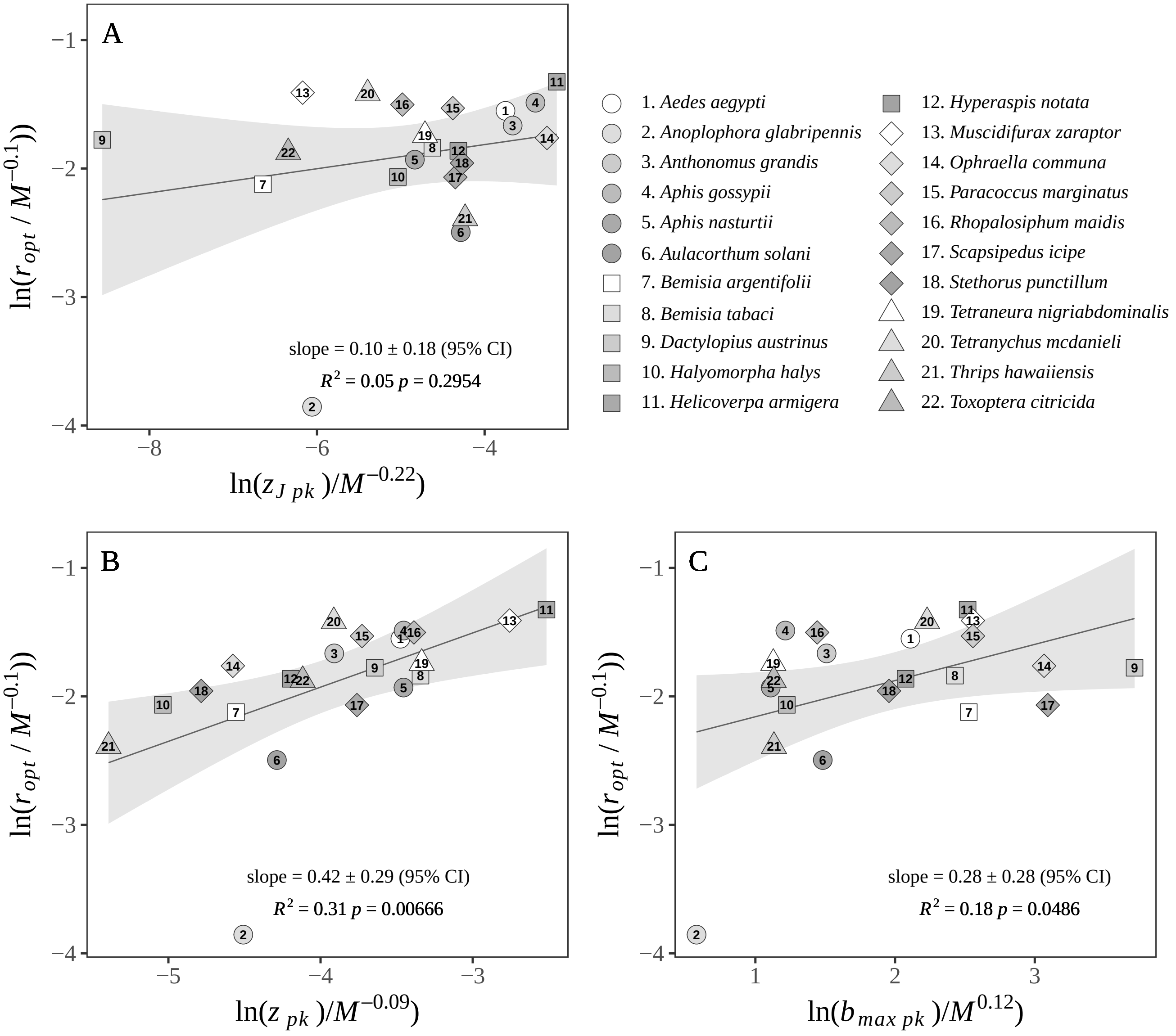

Supplementary Figure 2: Relationship between r_{opt} and peak A) juvenile mortality rate, B) adult mortality rate and C) fecundity rate. The lines are OLS regression (with 95% prediction bounds) fitted to the species' (n=22) log-transformed mass-corrected median r_{opt}s (symbols) plotted against their respective log-transformed mass-corrected median trait B_{pk}s.

1.3 Evidence of trait-level thermal adaptation

To test for trait-level thermal adaptation, we first analysed the relationships between trait T_{pk}s and latitudes (i.e., the geographical locations where each experimental species originated from). All traits' T_{pk}s declined with increasing latitude, which suggests that species, albeit weakly, are adapted to their local environments (SM Fig. 3), consistent with the patterns seen in other groups [3, 6]. Because environmental variables other than temperature also vary with latitude, we also analyzed the relationship between traits' T_{pk}s and rearing temperatures (i.e., the species' laboratory rearing temperatures; SM Fig. 4), and rearing temperatures and latitudes (SM Fig. 5). Trait T_{pk}s increased significantly with rearing temperatures (SM Fig. 4) and decreased significantly with increasing latitudes. These results suggest that species originating from warmer climates were reared under warmer temperatures in the lab. Together, these results indicate the existence of significant existing trait-level thermal adaption amongst the species used in the present study.

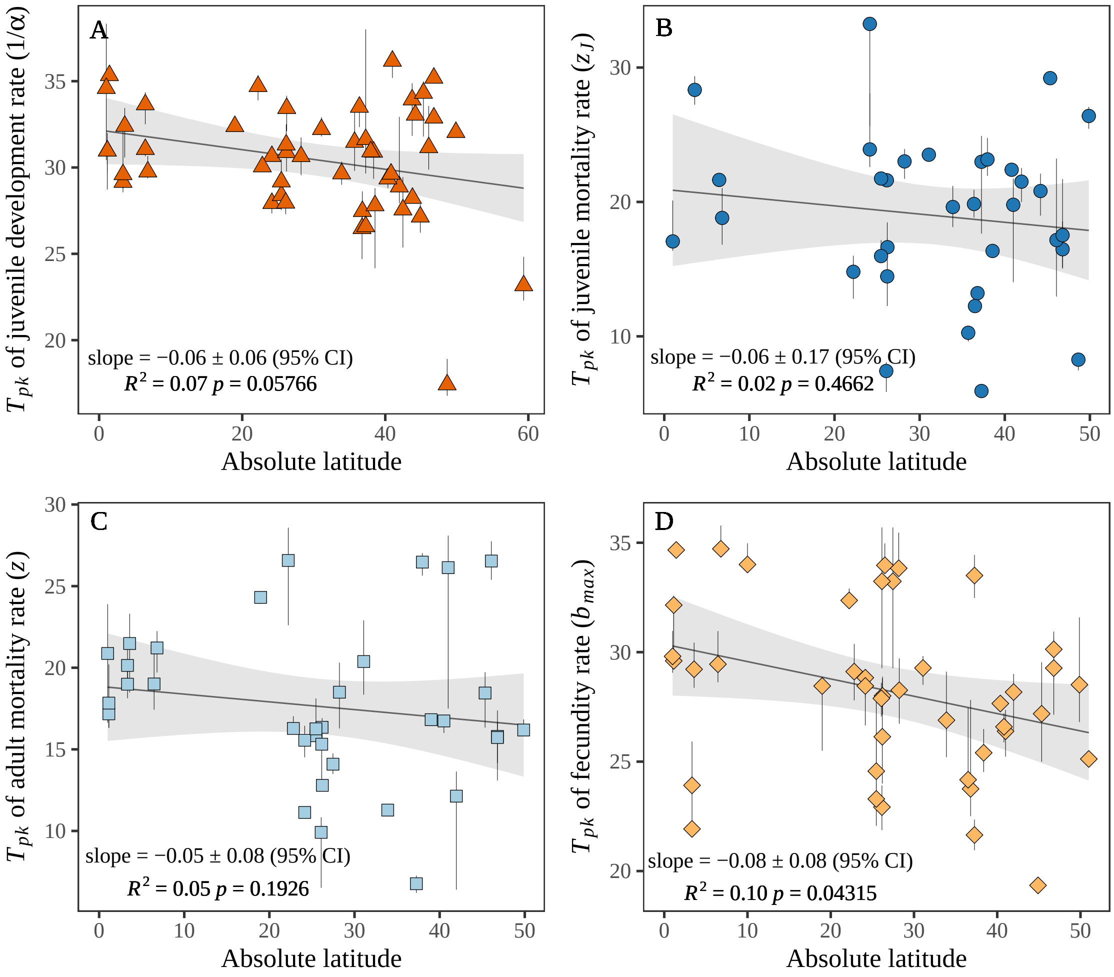

Supplementary Figure 3: Relationship between latitude and T_{pk}s of A) juvenile development rate ($1/\alpha$; n=49 species), B) juvenile mortality rate (n=34 species), C) adult mortality rate (n=34 species) and D) fecundity rate (n=44 species). The lines are OLS regression (with 95% prediction bounds) fitted to the species' median T_{pk} estimates (symbols) plotted against absolute latitude. Bootstrapping (residual resampling) was used to calculate 95% prediction bounds for each TPC (SM Figs. 7, 8, 9, 10), which also yielded the confidence intervals around the T_{pk} estimates shown.

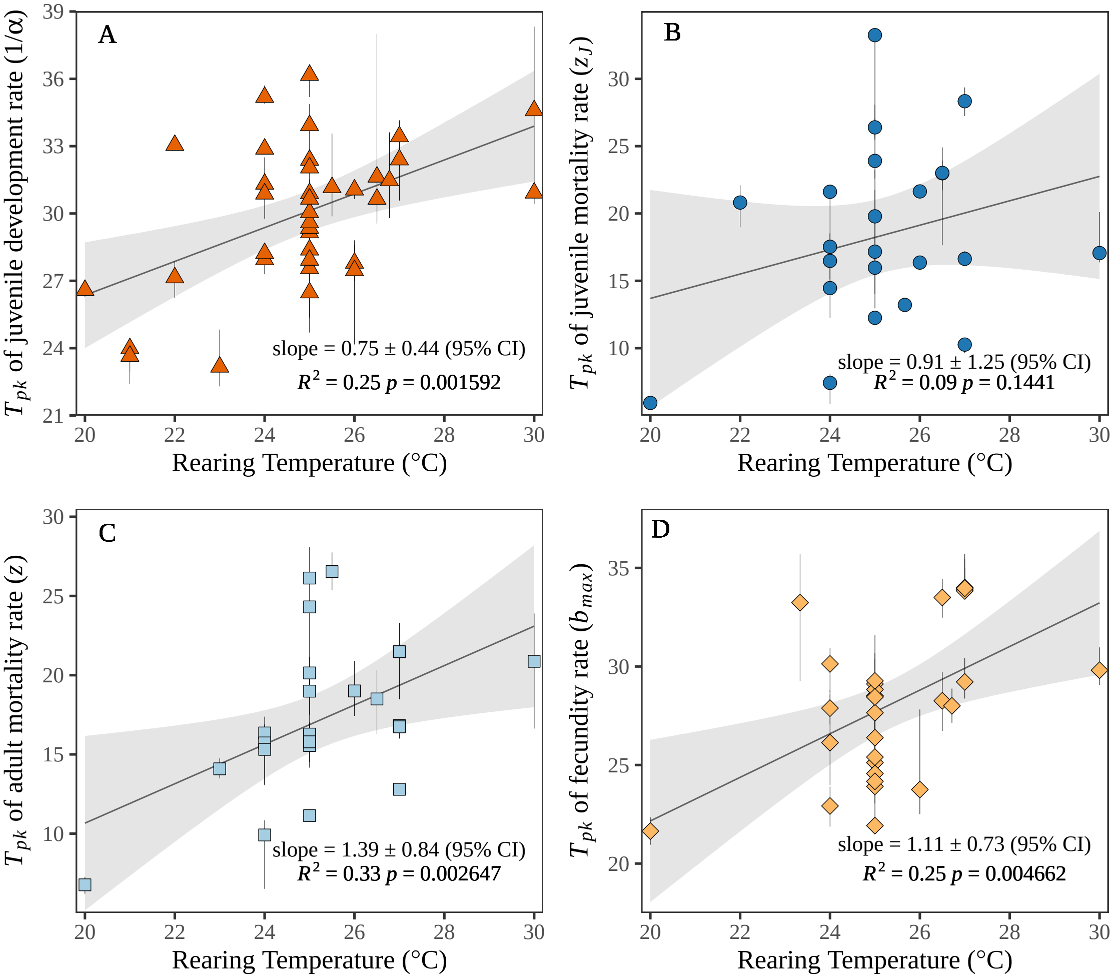

Supplementary Figure 4: Relationship between rearing temperature and T_{pk}s of A) juvenile development rate ($1/\alpha$; n=37 species), B) juvenile mortality rate (n=24 species), C) adult mortality rate (n=25 species) and D) fecundity rate (n=30 species). The lines are OLS regression (with 95% prediction bounds) fitted to the species' median T_{pk} estimates (symbols) plotted against rearing temperature. Bootstrapping (residual resampling) was used to calculate 95% prediction bounds for each TPC (SM Figs. 7, 8, 9, 10), which also yielded the confidence intervals around the T_{pk} estimates shown.

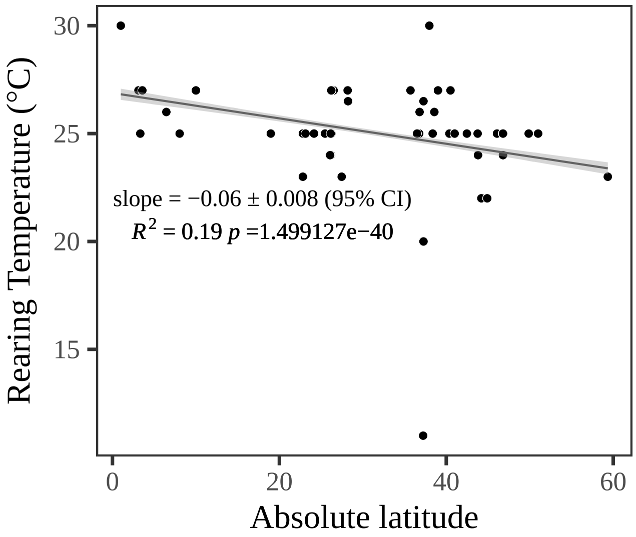

Supplementary Figure 5: Relationship between rearing temperature and absolute latitude. The lines are OLS regression (with 95% prediction bounds) fitted to the species' (n=45) rearing temperatures plotted against their respective latitudes.

1.4 Distributions of trait-level thermal sensitivities

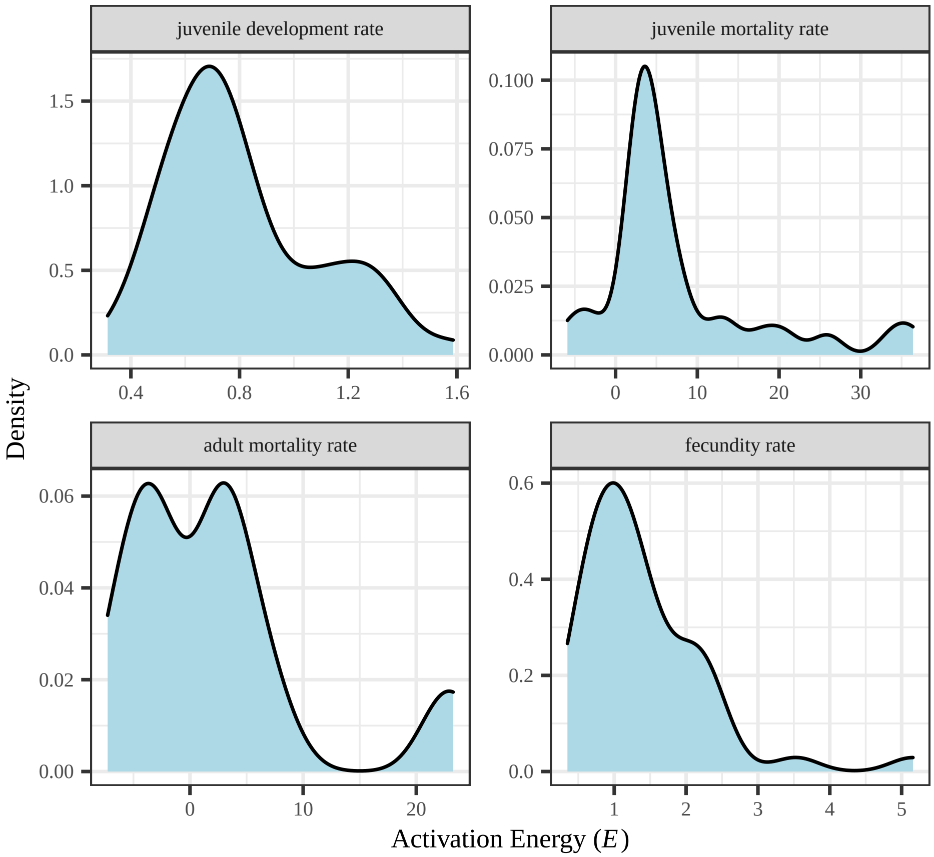

Supplementary Figure 6: Distribution of estimated activation energy values for all fitted traits and species.

1.5 Trait-level thermal performance curves

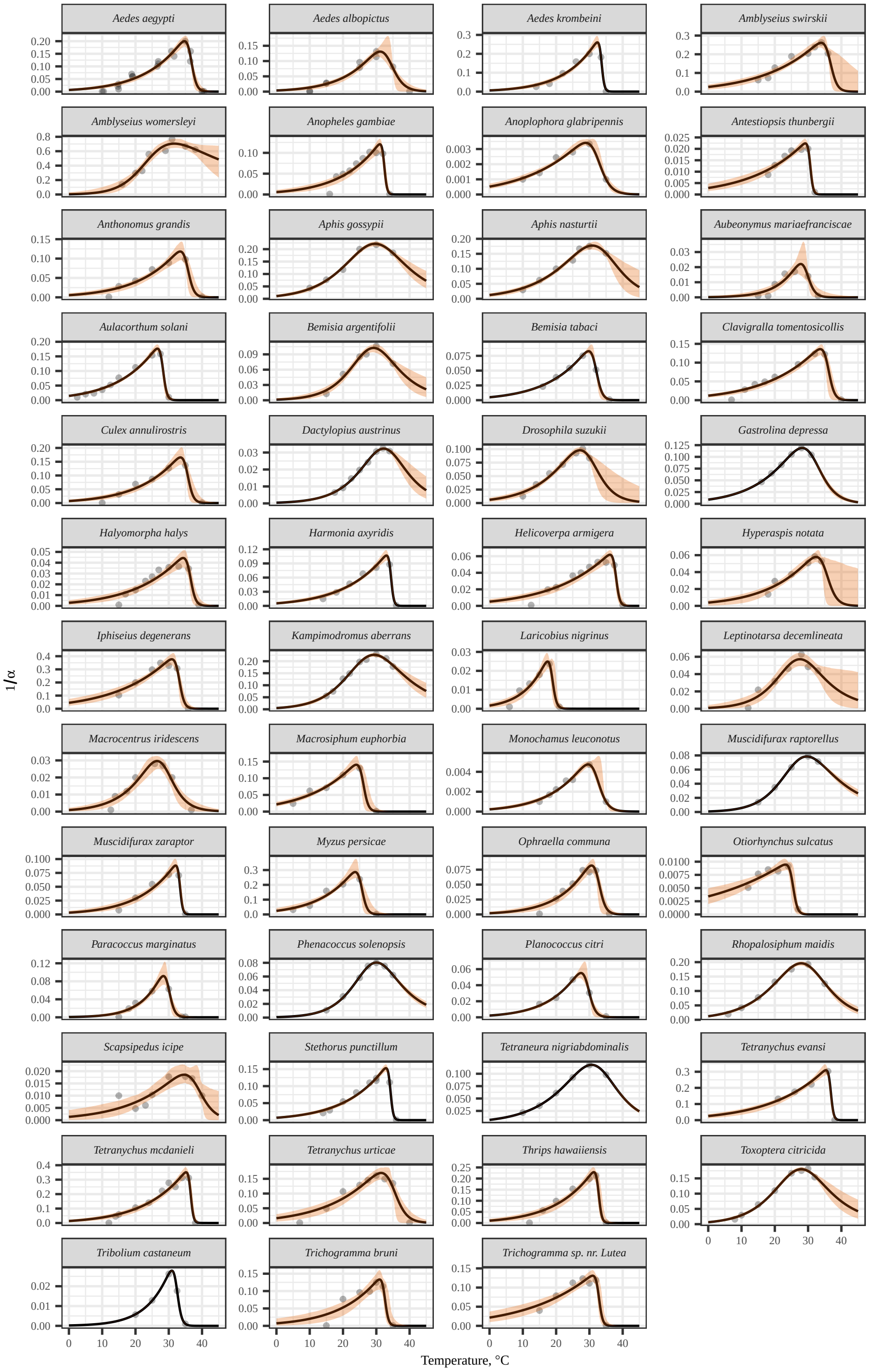

Supplementary Figure 7: Thermal Performance Curve fits for all species: Development Rate. Bootstrapping (residual resampling) was used to calculate 95% prediction bounds (shaded areas) for each TPC. These prediction bounds indicate statistical/data uncertainty arising from fitting the TPC model (Main text Equation 3) to TPC data where the peak was only captured through a single trait measurement at or close to that temperature. As such, this does not affect our results qualitatively.

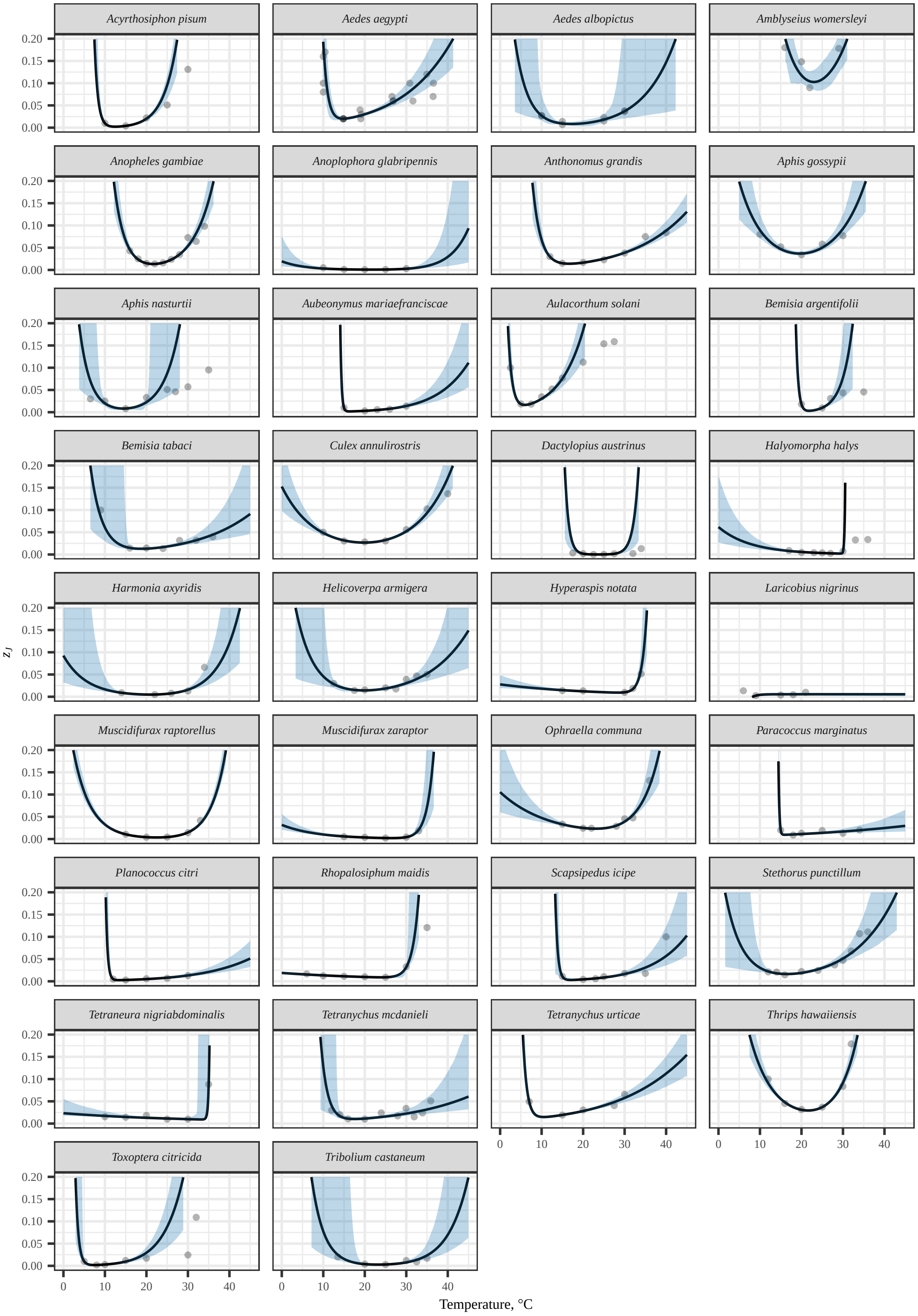

Supplementary Figure 8: Thermal Performance Curve fits for all species: Juvenile Mortality. Bootstrapping (residual resampling) was used to calculate 95% prediction bounds (shaded areas) for each TPC.

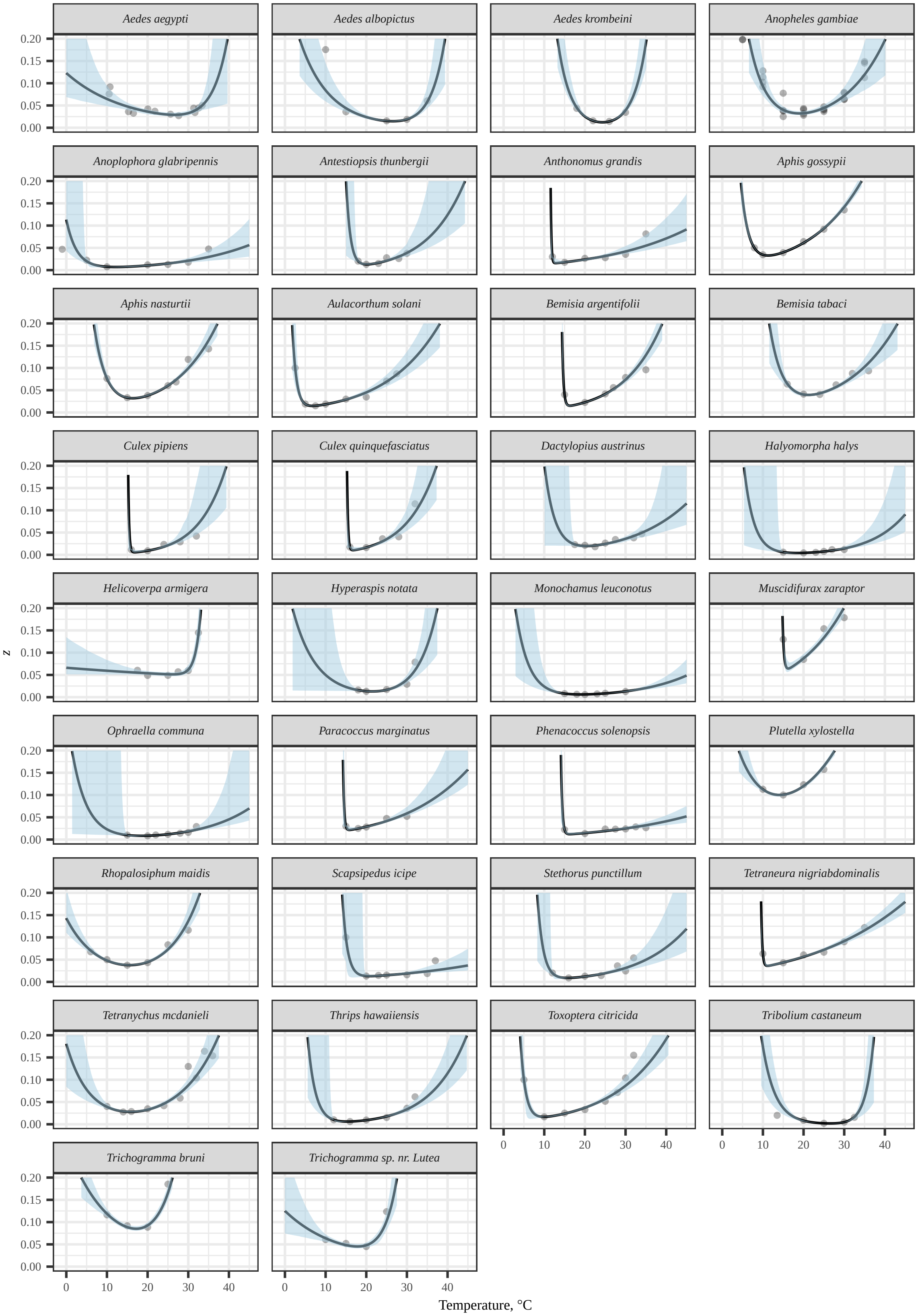

Supplementary Figure 9: Thermal Performance Curve fits for all species: Adult Mortality. Bootstrapping (residual resampling) was used to calculate 95% prediction bounds (shaded areas) for each TPC.

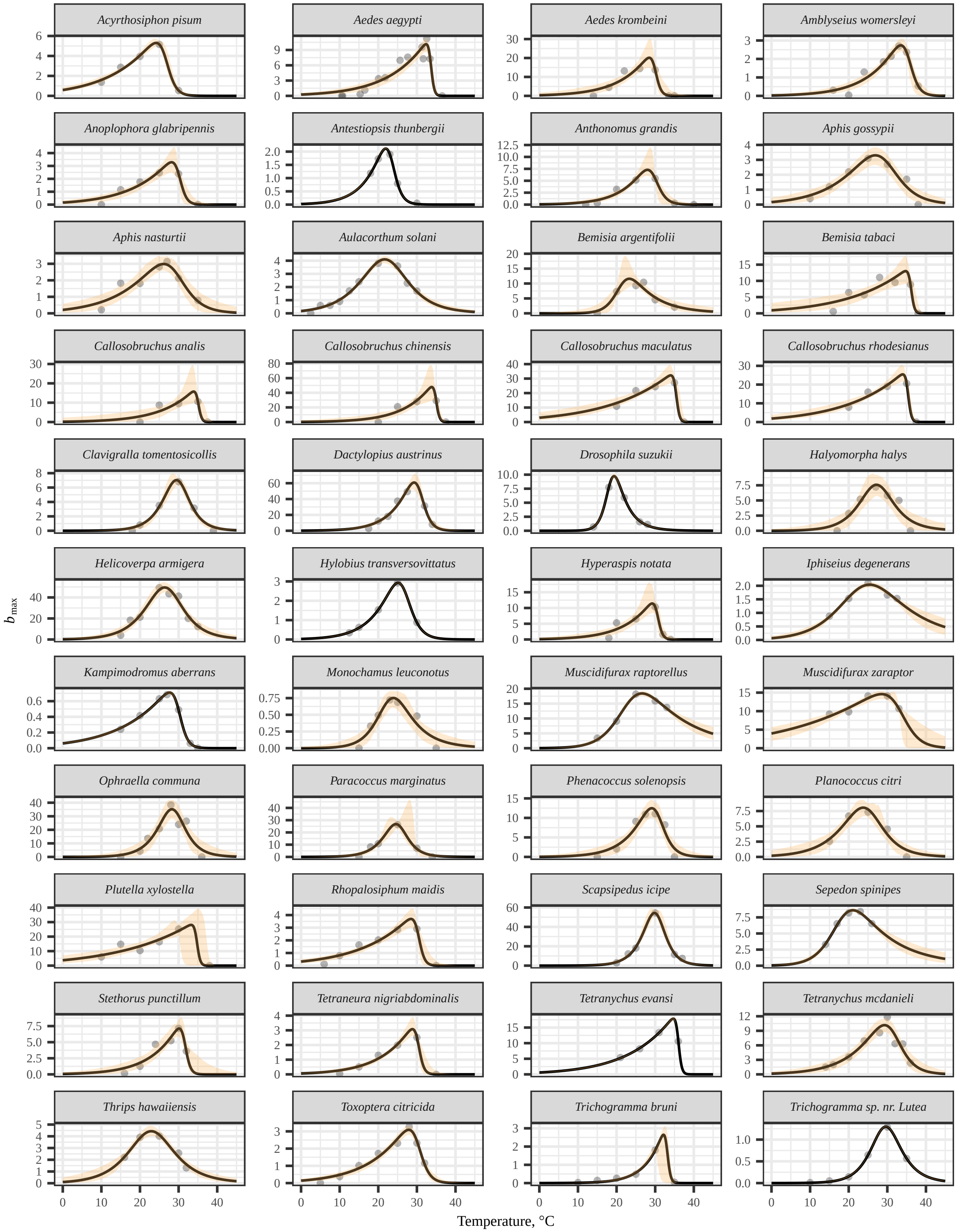

Supplementary Figure 10: Thermal Performance Curve fits for all species: Fecundity. Bootstrapping (residual resampling) was used to calculate 95% prediction bounds (shaded areas) for each TPC.

1.6 Species-level temperature dependencies of r_m

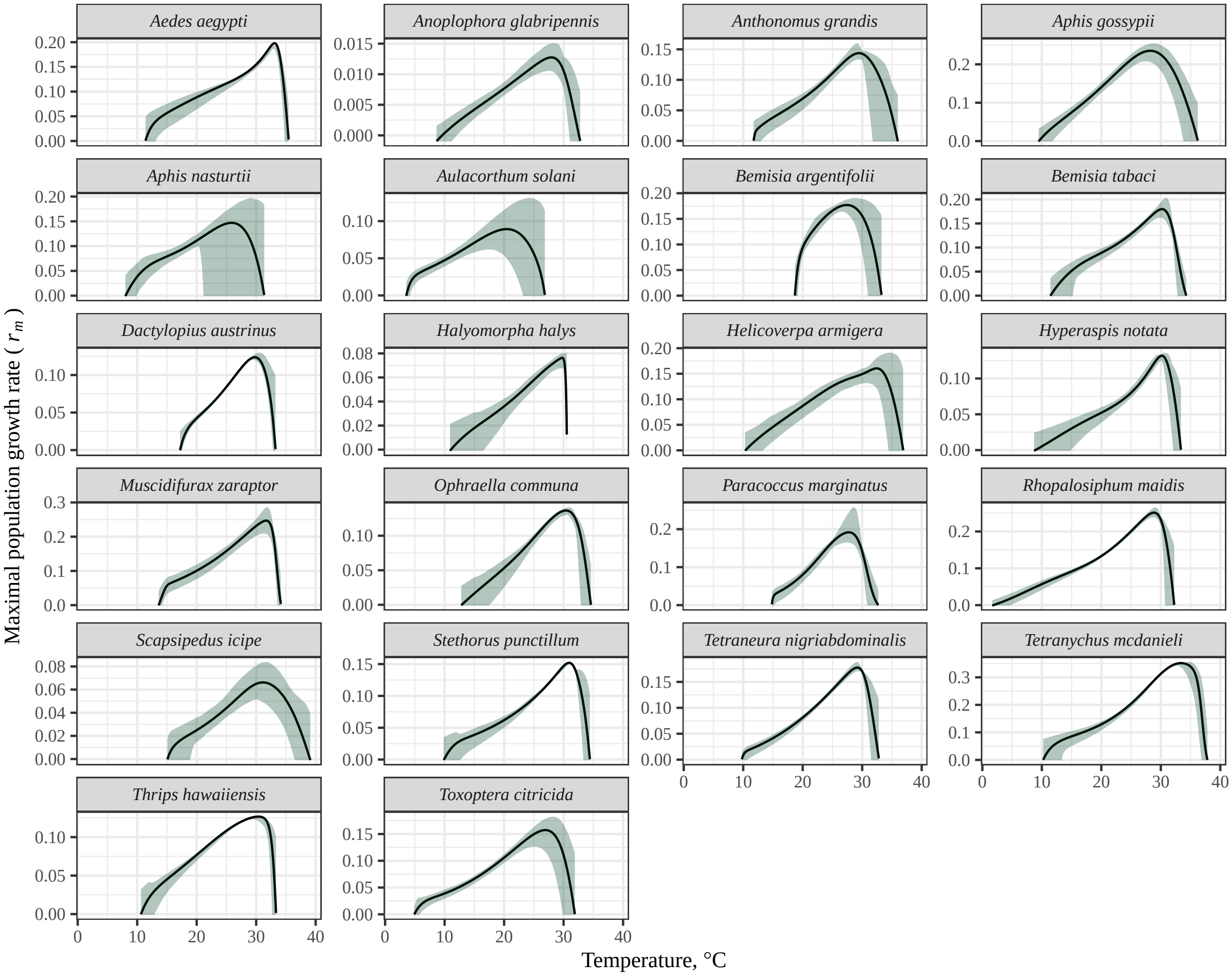

Supplementary Figure 11: Thermal Performance Curve fits for all species: r_m. Bootstrapping (residual resampling) was used to calculate 95% prediction bounds (shaded areas) for each TPC.

1.7 Sensitivity of the results to the parameterisation of fecundity loss rate (κ)

Fecundity typically declines over time, which can have significant impacts on the lifetime reproduction of individuals and therefore fitness. The rate at which fecundity declines with age (κ) may be temperature-dependent, but there appears to be practically no existing data on this for arthropods. Therefore, here we quantify the sensitivity of our theoretical predictions to changes in parameterisation of baseline fecundity loss rate (the normalisation constant, κ_0). Specifically, we re-evaluate our trait sensitivity analyses, as well as our calculation of selection gradient by varying κ_0 across two extreme values, around the value we have used to generate the main results (0.1). Supplementary Figure 12 shows how changing κ_0 affects the shape of the fecundity curve at any given temperature.

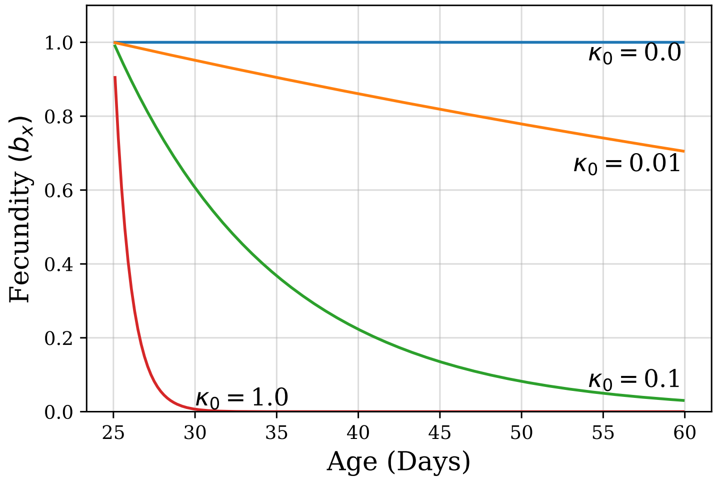

Supplementary Figure 12: Sensitivity of the fecundity TPC to changes in κ_0.

Supplementary Figure 13 shows that the TPC shape for κ remains qualitatively the same (for the two meaningful extreme values of κ_0):

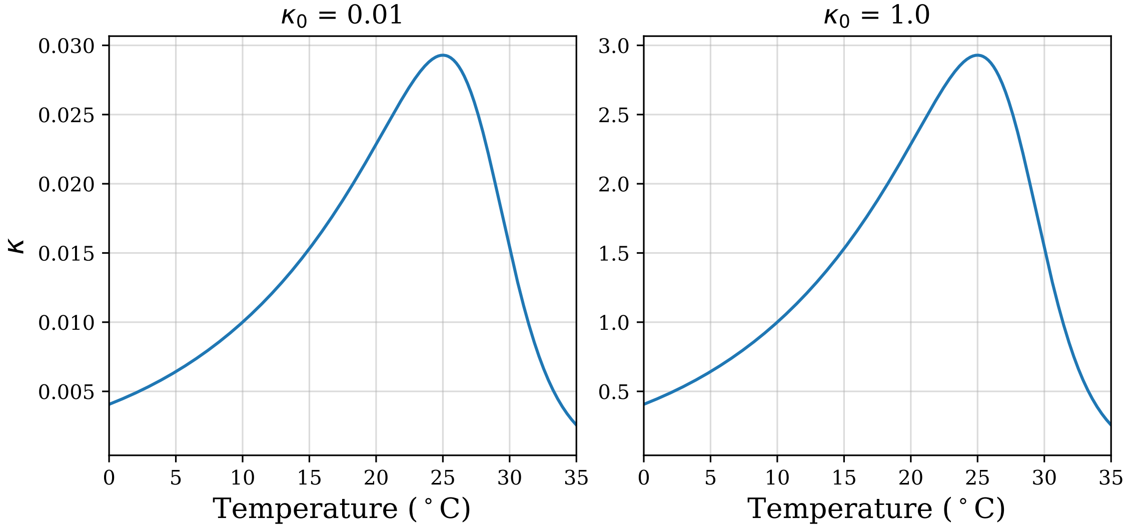

Supplementary Figure 13: Insensitivity of the κ curve to meaningfully extreme κ_0 values.

1.7.1 Effect on the trait sensitivity results

First, we re-evaluate the trait sensitivity analysis results (SM Fig. 14). As expected, in the case where baseline kappa (κ_0) is lower, maximum fecundity (b_{max}) becomes more important relative to κ, leaving the order of importance of the 5 traits the same as for the intermediate case (κ_0=0.1) upon which our main results are based.

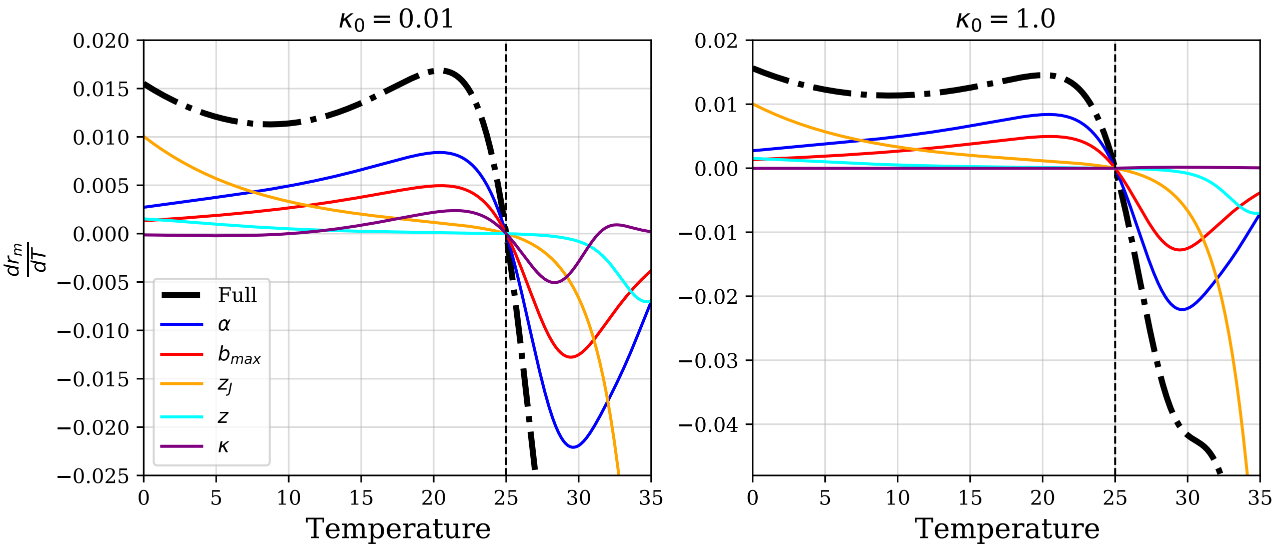

Supplementary Figure 14: Re-evaluation of the results of the trait sensitivity analysis

1.7.2 The selection gradients revisited

Next we re-evaluate the r_m TPC and selection gradients as above. We focus only on the dominant trait α because the order of the strengths of selection gradients is bound to remain unchanged due to the unchanged order of trait sensitivity irrespective of the κ_0 value (previous section). Supplementary Figure 15 shows that the selection gradient remains qualitatively unchanged, with overall r_m lower when κ_0 is high, as expected.

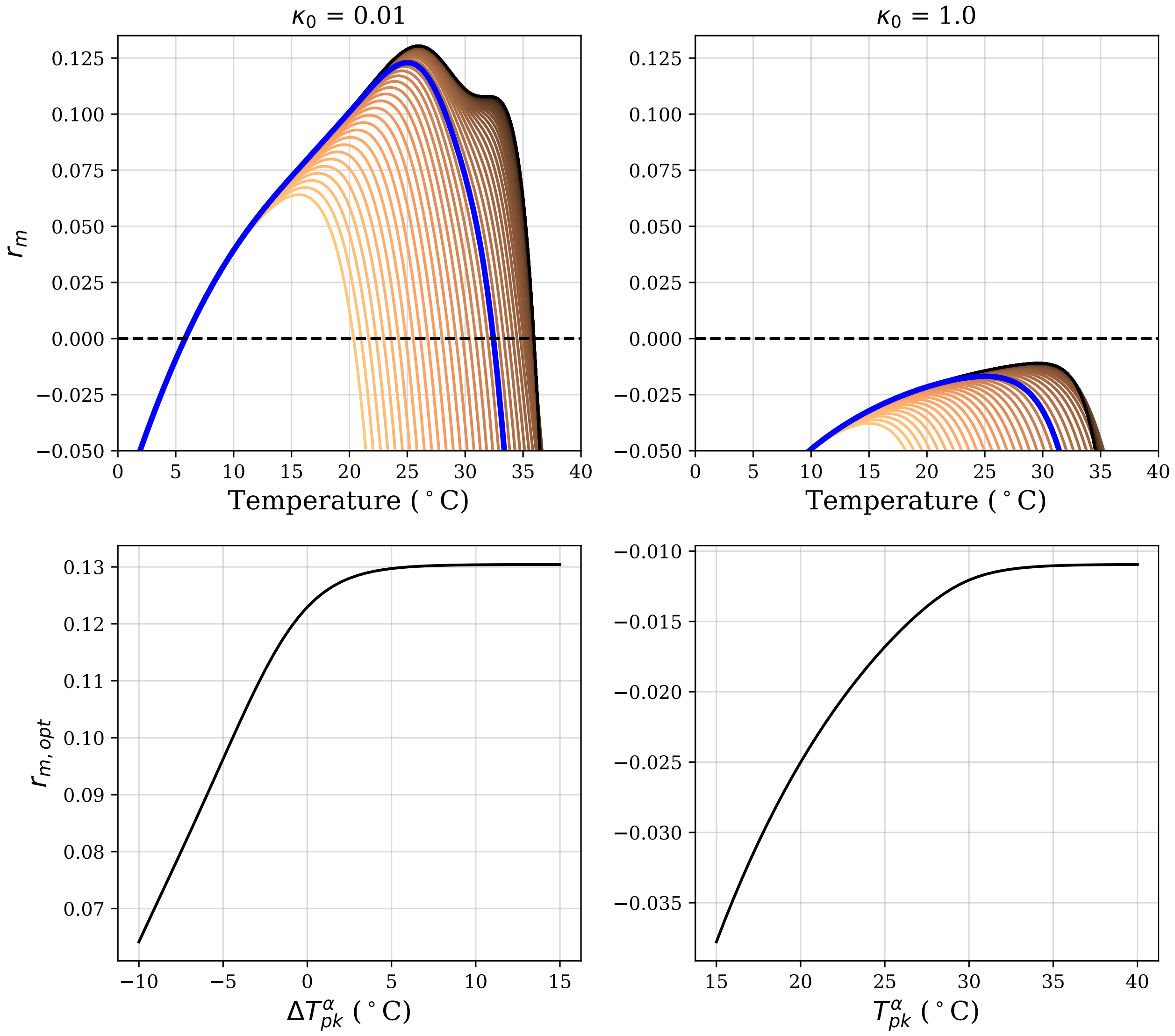

Supplementary Figure 15: Insensitivity of r_m selection gradient to changes in κ_0

1.8 Macroevolutionary patterns and phylogenetic constraints

Supplementary Table 1: Nucleotide sequences collected for each species from the SILVA (SSU and LSU) and Barcode of Life Data System (COI-5P) databases.

Species	SSU ID	LSU ID	COI-5P ID
Acyrthosiphon pisum	ABLF02002530.1.2075	F02004049.1.2297	-
Aedes aegypti	AAGE02033765.6222.8206	AAGE02025420.70.4046	CULSA016-19
Aedes albopictus	GCLM01041991.604.2545	MNAF02000533.14119.17736	ACMIP154-07
Aedes krombeini	-	-	-
Amblyseius swirskii	-	-	GBMNC68842-20
Amblyseius womersleyi	-	-	GBCH5643-13
Anopheles gambiae	AM157179.1.2015	LCWJ01002898.1.3298	CULSA066-19
Anoplophora glabripennis	-	-	GBMNE15612-21
Antestiopsis thunbergii	-	-	-
Anthonomus grandis	EU215423.9073.11005	EU215423.12455.16248	GBMIN12012-13
Aphis gossypii	-	-	ACEA143-14
Aphis nasturtii	-	-	ACEA530-14
Aubeonymus mariaefranciscae	-	-	-
Aulacorthum solani	AF487713.1.966	-	ACEA131-14
Bemisia argentifolii	-	-	-
Bemisia tabaci	GCZW01018184.195.2694	-	BTB002-12
Callosobruchus analis	-	-	GBCCH430-13
Callosobruchus chinensis	-	-	CSP030-09
Callosobruchus maculatus	GEUE01061439.889.2802	GEUD01155100.99.2572	GBCCH431-13
Callosobruchus rhodesianus	-	-	GBCL2570-06
Clavigralla tomentosicollis	GAJX01000019.410.2325	-	GBMNA17782-19
Culex annulirostris	-	-	GBMNC791-20
Culex pipiens	AY988445.1.1858	-	CULSA020-19
Culex quinquefasciatus	AAWU01003351.41983.43837	AAWU01047416.6583.8759	GBDP12712-12
Dactylopius austrinus	AY795538.1.608	-	-
Drosophila suzukii	AWUT01011932.32096.34063	AWUT01017126.2.3394	GBDPD245-14
Gastrolina depressa	-	-	GBMNA17939-19
Halyomorpha halys	GEDY01000115.149.2061	GBHT01004590.7.2044	AGIRI147-17
Harmonia axyridis	KP419116.1.1832	-	GBCL17655-14
Helicoverpa armigera	KT343378.1.1903	-	GBGL29849-19
Hylobius transversovittatus	-	-	GBCL3480-08
Hyperaspis notata	-	-	HEAUG012-12
Iphiseius degenerans	-	-	TZBCA378-07
Kampimodromus aberrans	-	-	-
Laricobius nigrinus	KP419143.1.1857	-	GBCL9856-12
Leptinotarsa decemlineata	GEEF01054810.5881.7790	-	FBCOP312-13
Macrocentrus iridescens	-	-	BBHEC285-09
Macrosiphum euphorbiae	-	-	ACEA250-14
Monochamus leuconotus	-	-	-
Muscidifurax raptorellus	-	-	-
Muscidifurax zaraptor	-	-	-
Myzus persicae	LXJY01000320.20922.22509	-	GBMNE22299-21
Ophraella communa	-	-	GBCCH11138-19
Otiorhynchus sulcatus	AF250084.1.1795	-	COLFD838-12
Paracoccus marginatus	FIZT01020513.6507.8206	-	GBMIN46559-16
Phenacoccus solenopsis	-	-	GBMHH29894-19
Planococcus citri	GAXF02029206.892.3340	-	GBMIN46549-16
Plutella xylostella	AHIO01004014.23148.25041	-	AACTA1852-20
Rhopalosiphum maidis	-	-	ACEA794-14
Scapsipedus icipe	-	-	GBMOR7430-19
Sepedon spinipes	-	-	FIDIP848-12
Stethorus punctillum	EF512328.1.1788	-	ASCMT030-11
Tetraneura nigriabdominalis	-	-	ASHMT220-11
Tetranychus evansi	AB926295.1.1858	-	GACAC126-12
Tetranychus mcdanieli	-	-	-
Tetranychus urticae	CAEY01001788.15245.16831	AY750693.1.2826	GACAC6465-19
Thrips hawaiiensis	-	-	AGIMP052-16
Toxoptera citricida	AY216697.1.2480	-	RDBA411-06
Tribolium castaneum	HM156711.1.1831	-	BIPR011-13
Trichogramma bruni	-	-	-
Trichogramma sp. nr. *Lutea*	-	-	KMPUH785-19

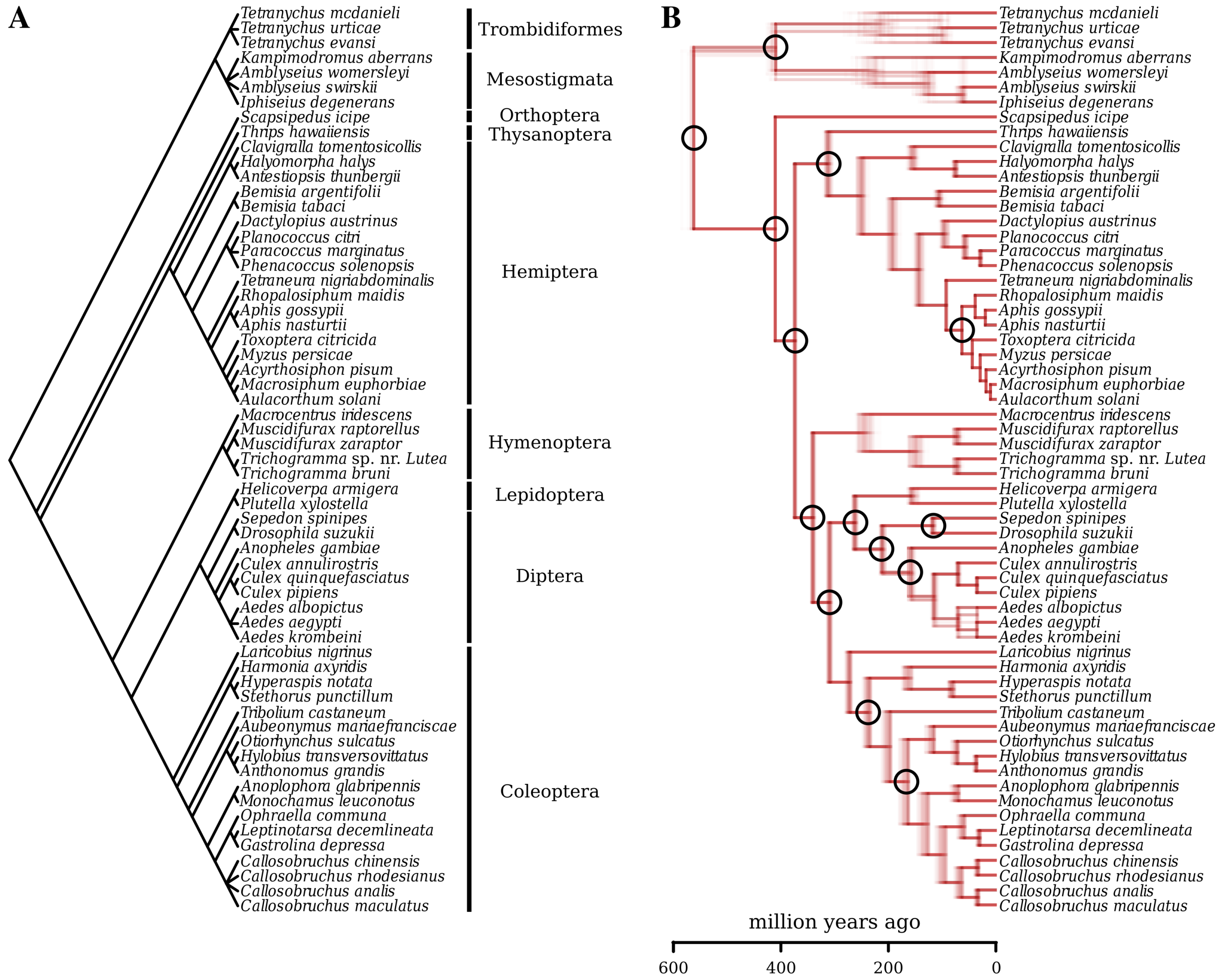

Supplementary Figure 16: A: The Open Tree of Life topology for the species in the study, with orders explicitly shown. Note that this topology includes some polytomies (e.g., see the *Callosobruchus* and *Tetranychus* clades). B: The final set of 100 time-calibrated trees overlaid on top of each other. Differences in both topology and branch lengths can be observed. Some polytomies from panel A were objectively resolved (e.g., the *Callosobruchus* clade) based on the concatenated sequence alignment. In contrast, where sequence data were completely missing for at least one species (e.g., the *Tetranychus* clade), polytomies were randomly resolved. Nodes whose age was obtained from the TimeTree database are marked with a circle. The two panels were plotted with the ape [7] (v.5.6-2) and phytools R packages [8] (v.1.2-0), respectively.

References

[1] Savage, V. M., Deeds, E. J. & Fontana, W. Sizing up allometric scaling theory. *PLoS computational biology* **4**, e1000171 (2008).

[2] Savage, V. M. *et al.* Effects of body size and temperature on population growth. *Am. Nat.* **163**, 429–41 (2004).

[3] Kontopoulos, D.-G. *et al.* Phytoplankton thermal responses adapt in the absence of hard thermodynamic constraints. *Evolution* **74**, 775–790 (2020).

[4] Frazier, M., Huey, R. B. & Berrigan, D. Thermodynamics constrains the evolution of insect population growth rates:"warmer is better". *Am. Nat.* **168**, 512–520 (2006).

[5] Pawar, S., Dell, A. I., Savage, V. M. & Knies, J. L. Real versus Artificial Variation in the Thermal Sensitivity of Biological Traits. *Am. Nat.* **187**, E41–E52 (2016).

[6] Huey, R. B. *Evolutionary physiology of insect thermal adaptation to cold environments*, 223–241 (Cambridge University Press, 2010).

[7] Paradis, E. & Schliep, K. ape 5.0: an environment for modern phylogenetics and evolutionary analyses in R. *Bioinformatics* **35**, 526–528 (2019).

[8] Revell, L. J. phytools: an R package for phylogenetic comparative biology (and other things). *Methods in Ecology and Evolution* **3**, 217–223 (2012).
