# Supplementary material for: Variation in temperature of peak trait performance constrains adaptation of arthropod populations to climatic warming
Source: Nat Ecol Evol. 2024 Jan 25;8(3):500–10. doi: 10.1038/s41559-023-02301-8 (PMC10927549; doi:10.1038/s41559-023-02301-8)
Supplement: Supplementary file 2 — Reporting Summary [file 41559_2023_2301_MOESM2_ESM.pdf]

nature portfolio

Corresponding author(s): Paul Huxley, Samraat Pawar, Lauren Cator

Last updated by author(s): Nov 15, 2023

Reporting Summary

Nature Portfolio wishes to improve the reproducibility of the work that we publish. This form provides structure for consistency and transparency in reporting. For further information on Nature Portfolio policies, see our Editorial Policies and the Editorial Policy Checklist.

Statistics

For all statistical analyses, confirm that the following items are present in the figure legend, table legend, main text, or Methods section.

n/a	Confirmed	
☐	☒	The exact sample size (n) for each experimental group/condition, given as a discrete number and unit of measurement
☐	☒	A statement on whether measurements were taken from distinct samples or whether the same sample was measured repeatedly
☐	☒	The statistical test(s) used AND whether they are one- or two-sided<br>*Only common tests should be described solely by name; describe more complex techniques in the Methods section.*
☐	☒	A description of all covariates tested
☐	☒	A description of any assumptions or corrections, such as tests of normality and adjustment for multiple comparisons
☐	☒	A full description of the statistical parameters including central tendency (e.g. means) or other basic estimates (e.g. regression coefficient) AND variation (e.g. standard deviation) or associated estimates of uncertainty (e.g. confidence intervals)
☐	☒	For null hypothesis testing, the test statistic (e.g. F, t, r) with confidence intervals, effect sizes, degrees of freedom and P value noted<br>*Give P values as exact values whenever suitable.*
☐	☒	For Bayesian analysis, information on the choice of priors and Markov chain Monte Carlo settings
☒	☐	For hierarchical and complex designs, identification of the appropriate level for tests and full reporting of outcomes
☒	☐	Estimates of effect sizes (e.g. Cohen's d, Pearson's r), indicating how they were calculated

Our web collection on statistics for biologists contains articles on many of the points above.

Software and code

Policy information about availability of computer code

Data collection: Relevant temperature-trait responses were extracted manually from the text or tables of published literature, or were read from the figures using WebPlotDigitizer (https://automeris.io/WebPlotDigitizer/).

Data analysis: All data and code for reproducing the study's analyses can be found at: https://github.com/EcoEngLab/TraitMismatchPaper-main.git. Our global dataset on arthropods is also available as a Source data file and in the fully open VecTraits database (https://vectorbyte.crc.nd.edu/vectraits-explorer).

For manuscripts utilizing custom algorithms or software that are central to the research but not yet described in published literature, software must be made available to editors and reviewers. We strongly encourage code deposition in a community repository (e.g. GitHub). See the Nature Portfolio guidelines for submitting code & software for further information.

Data

Policy information about availability of data

All manuscripts must include a data availability statement. This statement should provide the following information, where applicable:

- Accession codes, unique identifiers, or web links for publicly available datasets
- A description of any restrictions on data availability
- For clinical datasets or third party data, please ensure that the statement adheres to our policy

All data used and generated in the study can be found at: https://github.com/EcoEngLab/TraitMismatchPaper-main.git. Our global dataset on arthropods is also available as a Source data file and in the fully open VecTraits database (https://vectorbyte.crc.nd.edu/vectraits-explorer).

Research involving human participants, their data, or biological material

Policy information about studies with human participants or human data. See also policy information about sex, gender (identity/presentation), and sexual orientation and race, ethnicity and racism.

Reporting on sex and gender	N/A
Reporting on race, ethnicity, or other socially relevant groupings	N/A
Population characteristics	N/A
Recruitment	N/A
Ethics oversight	N/A

Note that full information on the approval of the study protocol must also be provided in the manuscript.

Field-specific reporting

Please select the one below that is the best fit for your research. If you are not sure, read the appropriate sections before making your selection.

☐ Life sciences ☐ Behavioural & social sciences ☒ Ecological, evolutionary & environmental sciences

For a reference copy of the document with all sections, see nature.com/documents/nr-reporting-summary-flat.pdf

Ecological, evolutionary & environmental sciences study design

All studies must disclose on these points even when the disclosure is negative.

Study description

We performed an extensive literature search to collate existing data on the TPCs of individual traits across arthropod taxa. Each species- and trait-specific thermal response dataset was fitted to the appropriate TPC equation (Eqn 3 or its inverse (Fig. 1A & B) using NLLS using the rTPC pipeline (Padfield 2021; Huxley et al. 2022). Bootstrapping (residual resampling) was used to calculate 95% prediction bounds for each TPC, which also yielded the confidence intervals around each Tpk and peak trait value (Bpk) estimate. These trait value estimates were body mass-corrected to adjust for how the temperature-dependence of rm emerges from the temperature-dependencies of its underlying traits, which, in turn, are dependent on the relationships between body size and metabolic rate in individual organisms (Savage 2004). If fresh mass (mg, M in Main text Fig.4; Supplementary figures 1 & 2) for a particular species was not provided in the original study, we used mass estimates from other studies on that or a closely related species. To test the hotter-better-pattern across taxa, the Bpk values were estimated by fitting either Eqn 3 (for alpha) or its inverse (for zJ and z) for individual species' TPCs using NLLS.

To examine the macroevolutionary patterns of the Tpks of the four main traits of this study (alpha, bmax, zJ, and z), we first extracted the phylogenetic topology of all the species in our dataset from the Open Tree of Life (OTL; v.13.4) using the rotl R package (v.3.0.12). Given that the OTL topology (Supplementary Fig. 16A) included a few polytomies, we also collected publicly available nucleotide sequences (where available) of: i) the 5' region of the cytochrome c oxidase subunit I gene (COI-5P); ii) the small subunit rRNA gene (SSU); and iii) the large subunit rRNA gene (LSU). COI-5P sequences were obtained from the Barcode of Life Data System database whereas SSU and LSU sequences were extracted from the SILVA database (Supplementary Table 1).

Research sample

Our global dataset on arthropods is comprised of mean values for relevant traits across temperatures. These values were extracted from the text or tables, or were read from the figures of published studies using WebPlotDigitizer (Rohatgi 2020). This dataset can be found at: https://github.com/EcoEngLab/TraitMismatchPaper-main.git. It is also available as a Source data file and in the fully open VecTraits database (https://vectorbyte.crc.nd.edu/vectraits-explorer).

To examine the macroevolutionary patterns of the Tpks of the four main traits of this study (alpha, bmax, zJ, and z), we first extracted the phylogenetic topology of all the species in our dataset from the Open Tree of Life (OTL; v.13.4) using the rotl R package (v.3.0.12).

	Given that the OTL topology (Supplementary Fig. 16A) included a few polytomies, we also collected publicly available nucleotide sequences (where available) of: i) the 5' region of the cytochrome c oxidase subunit I gene (COI-5P); ii) the small subunit rRNA gene (SSU); and iii) the large subunit rRNA gene (LSU). COI-5P sequences were obtained from the Barcode of Life Data System database whereas SSU and LSU sequences were extracted from the SILVA database (Supplementary Table 1).
Sampling strategy	Values (means) of relevant traits across temperatures at were extracted from the text or tables, or were read from the figures of published studies using WebPlotDigitizer (Rohatgi 2020).
Data collection	PH and MN performed an extensive literature search to collate existing data on the thermal performance curves of individual traits across arthropod taxa.
Timing and spatial scale	We searched for publications up to July 2022 using Google Scholar's advanced search, using Boolean operators (e.g., life history AND pest OR vector AND temper*), without language restrictions. Additional searches were also made by including species' names in the search string to improve the detection of publications on under-represented groups.
Data exclusions	For some species, multiple data sets were available so we only included the study that provided the most complete data (i.e., the highest number of traits measured). We excluded all field studies, and also lab studies where traits had been measured over narrow (<10 degrees Celsius) temperature ranges (that prevented reliable TPC model fitting). We also excluded species from our analysis of physiological mismatches if TPC data for at least two traits were not available for them.
Reproducibility	All data and code used and generated in the study can be found at: https://github.com/EcoEngLab/TraitMismatchPaper-main.git. Our global dataset on arthropods is also available as a Source data file and in the fully open VecTraits database (https://vectorbyte.crc.nd.edu/vectraits-explorer).
Randomization	Organisms were allocated to groups based on their scientific species names.
Blinding	The objective was to collate all available published data on the temperature-dependence of arthropod fitness traits. Therefore, blinding was not applicable to this study.

Did the study involve field work? ☐ Yes ☒ No

Reporting for specific materials, systems and methods

We require information from authors about some types of materials, experimental systems and methods used in many studies. Here, indicate whether each material, system or method listed is relevant to your study. If you are not sure if a list item applies to your research, read the appropriate section before selecting a response.

Materials & experimental systems

n/a	Involved in the study	
☒	☐	Antibodies
☒	☐	Eukaryotic cell lines
☒	☐	Palaeontology and archaeology
☒	☐	Animals and other organisms
☒	☐	Clinical data
☒	☐	Dual use research of concern
☒	☐	Plants

Methods

n/a	Involved in the study	
☒	☐	ChIP-seq
☒	☐	Flow cytometry
☒	☐	MRI-based neuroimaging
