# Supplementary material for: Variation in temperature of peak trait performance constrains adaptation of arthropod populations to climatic warming
Source: Nat Ecol Evol. 2024 Jan 25;8(3):500–10. doi: 10.1038/s41559-023-02301-8 (PMC10927549; doi:10.1038/s41559-023-02301-8)
Supplement: Supplementary file 4 — Auxiliary Supplementary File (Appendix 2) containing detail and code of how Main text equation 2 was derived. [file 41559_2023_2301_MOESM4_ESM.pdf]

Auxiliary Supplementary File (Appendix 2) for:

Variation in temperature of peak trait performance constrains adaptation of arthropod populations to climatic warming

In [1]:

```python
#Load some modules etc
import matplotlib.pyplot as plt
plt.rcParams.update(plt.rcParamsDefault) # Reset plots to default style
plt.rcParams['font.family'] = 'serif'
plt.rcParams['axes.grid'] = 'true'
plt.rcParams['axes.axisbelow'] = True
plt.rcParams['grid.alpha'] = .45
# plt.rcParams['axes.xmargin'] = 0
# plt.rcParams['axes.xmargin'] = 0
plt.rcParams['figure.figsize'] = [10/2.54, 8/2.54]


from sympy import *
import scipy as sc
import numpy as np
import warnings
warnings.filterwarnings('ignore')
init_printing()
%matplotlib inline
```

Model for arthropod maximum population growth rate (fitness)

In age-structured populations, the expected reproductive success of an individual can be described using the continuous form of the Euler-Lotka equation:

$$\int_{\alpha}^{\infty} \mathrm{e}^{-r_m x} l_x b_x \, \mathrm{d}x = 1$$

where r_m is the maximal population growth rate, a is the age of first reproduction, l_x is the age-specific survivorship, and b_x is the age-specific fecundity. Solving this equation gives r_m as a function of life-history parameters (the life-history traits).

In [2]:

```python
x, l_a, b_max, alpha, z, z_J, kappa, T, M_0, K, t = var('x l_a b_max alpha z z_J kappa T M_0 K t',real=True, po

r_m  = var('r_m', real = True) # r_m can be negative
```

Because a Type III survivorship curve within life stages (which assumes a constant mortality rate) is a resonable approximation for arthropods (Amarasekare and Savage, 2012), given an instantaneous mortality rate, z, age-specific survivorship, l_x, declines exponentially with age and can be modeled as:

$$l_x = l_\alpha \mathrm{e}^{-z(x-\alpha)} \tag{1}$$

Here, l_α is the proportion of eggs surviving to adulthood (age α), which, assuming a fixed instantaneous mortality rate across all juvenile stage, z_J, can be modelled as:

$$l_\alpha = \mathrm{e}^{-\int_0^\alpha z_J \, \mathrm{d}x} = \mathrm{e}^{-z_J \alpha} \tag{2}$$

In [3]:

```python
l_a = exp(-integrate(z_J,(x,0,alpha))); l_a
```

Out[3]: $e^{-\alpha z_J}$

This, when substituted into the l_x equation then gives

$$l_x = \mathrm{e}^{-(z_J \alpha + z(x-\alpha))} \tag{3}$$

In [4]:

```python
l_x = l_a * exp(-z*(x - alpha)); simplify(l_x)
```

Out[4]: $e^{-\alpha z_J + z(\alpha - x)}$

(where $x \geq \alpha$).

Next, age-specific fecundity b_x is expected to reach a peak, b_{max}, shortly after maturation and then decline gradually with age. We model this using an exponential function

$$b_x = b_{max} \mathrm{e}^{\kappa(\alpha - x)}$$

where we assume that fecundity peaks at the age of first reproduction, which occurs immediately at the time of adult emergence (α), and κ is a shape parameter (the fecundity loss rate in day^{-1}) that controls the spread of the fecundity schedule, that is, the rate of decline in fecundity after its peak. It is also possible that there is a systematic temperature-dependence of the delay between α and the age of peak fecundity. This can be modelled using a shifted exponential or gompertz equation, but is outside the scope of the current study. The figures below show the shape of the b_x model.

In [5]:

```python
b_x = b_max * exp(-kappa*(x - alpha)); simplify(b_x)
```

Out[5]: $b_{max} e^{\kappa(\alpha - x)}$

We can now visualize these functions.

In [6]:

```python
#assign parameter values

z_J_par = .05 #1/day
z_par = .03 #1/day
alp_par = 25. #days
b_max_par = 1. #individuals/(individual * day)
kap_par = .1 #1/day
```

In [7]:

```python
#Numerically evaluate
x_vec = np.arange(0, 60, 0.1) #vector of ages

l_x_lam = lambdify((z_J, z, alpha,x), l_x, np) # Use lambidfy to speed up
l_x_vec = l_x_lam(z_J_par, z_par, alp_par, x_vec[x_vec>alp_par])

fig = plt.figure(); ax = fig.add_subplot(111)
ax.plot(x_vec[x_vec>alp_par], l_x_vec)
ax.set_xlabel('Age (days)', fontsize=14)
ax.set_ylabel('Survivorship ($l_x$)', fontsize=14)
ax.text(np.mean(x_vec[x_vec>alp_par]), np.amax(l_x_vec),
        '$z_J = ' + str(z_J_par)+'$ \n' +
        '$z = ' + str(z_par)+'$ \n' +
        r'$\alpha = ' + str(alp_par)+'$ \n',
        horizontalalignment='left', verticalalignment='top', fontsize=15)

#plt.savefig('../results/Figs/lxModel.pdf', bbox_inches='tight')
```

Out[7]: `Text(42.5, 0.28564657045289016, '$z_J = 0.05$ \n$z = 0.03$ \n$\\alpha = 25.0$ \n')`

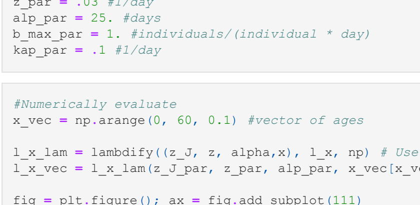

In [8]:

```python
fig = plt.figure(); ax = fig.add_subplot(111)
b_x_lam = lambdify((b_max, kappa, alpha, x), b_x, np) #use lambidy to speed up
b_x_vec = b_x_lam(b_max_par, kap_par, alp_par, x_vec[x_vec>alp_par])

ax.plot(x_vec[x_vec>alp_par], b_x_vec);
ax.set_xlabel('Age (days)', fontsize=14);
ax.set_ylabel('Fecundity ($b_x$)', fontsize=14)
ax.text(np.mean(x_vec[x_vec>alp_par]), np.amax(b_x_vec)/2,
        r'$\kappa = ' + str(kap_par)+'$ \n'+
        r'$\alpha = ' + str(alp_par)+'$ \n' +
        r'$b_{max} = ' + str(b_max_par)+'$ \n',
        horizontalalignment='left', verticalalignment='top', fontsize=15)

#plt.savefig('../results/Figs/bxModel.pdf')
```

Out[8]: `Text(42.5, 0.49502491687458394, '$\\kappa = 0.1$ \n$\\alpha = 25.0$ \n$b_{max} = 1.0$ \n')`

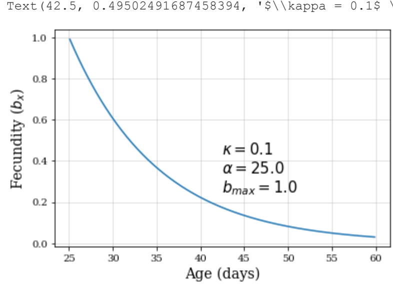

In [9]:

```python
fig = plt.figure(); ax = fig.add_subplot(111) #now plot lxmx

ax.plot(x_vec[x_vec>alp_par], b_x_vec*l_x_vec);
ax.set_xlabel('Age (days)', fontsize=14);
ax.set_ylabel('$l_x b_x$', fontsize=14)

#plt.savefig('../results/Figs/lxbxModel.pdf', bbox_inches='tight')
```

Out[9]: `Text(0, 0.5, '$l_x b_x$')`

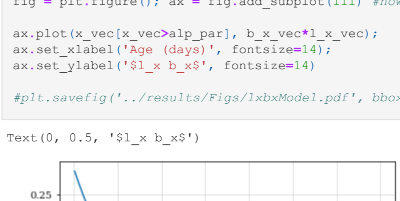

Next, substituting the l_x and b_x models into the Euler-Lotka Equation, we get

$$\int_{\alpha}^{\infty} b_{max} \mathrm{e}^{-\alpha z_J + \kappa(\alpha - x) - rx + z(\alpha - x)} \, \mathrm{d}x = 1$$

i.e.,

$$b_{max} \mathrm{e}^{-\alpha z_J} \int_{\alpha}^{\infty} \mathrm{e}^{-rx + (\kappa + z)(\alpha - x)} \, \mathrm{d}x = 1$$

In [10]:

```python
EuLo = exp(-r_m * x) * l_x * b_x; simplify(EuLo)
```

Out[10]: $b_{max} e^{-\alpha z_J + \kappa(\alpha - x) - r_m x + z(\alpha - x)}$

And evaluating the integral produces after simplification:

$$\frac{b_{max} e^{-\alpha(r + z_J)}}{\kappa + r + z} = 1 \tag{4}$$

In [11]:

```python
# Integrate EuLo (sagemath used to integrate(EuLo, x, alpha, infinity), with positivity constraints on all para
EuLo_int = b_max*exp(-alpha*z_J)/((kappa + r_m)*exp(alpha*r_m) + z*exp(alpha*r_m)); simplify(EuLo_int)
```

Out[11]: $\frac{b_{max} e^{-\alpha(r_m + z_J)}}{\kappa + r_m + z}$

Solving this for r_m gives:

$$r_m = \frac{1}{\alpha}\left(-\alpha(\kappa + z) + \operatorname{LambertW}\left(\alpha b_{max} e^{\alpha(\kappa + z - z_J)}\right)\right) \tag{5}$$

In [12]:

```python
r_SP = solve(EuLo_int-1,r_m); r_SP = simplify(r_SP[0]); r_SP
```

Out[12]: $\frac{-\alpha(\kappa + z) + W\left(\alpha b_{max} e^{\alpha(\kappa + z - z_J)}\right)}{\alpha}$

The LambertW function part is difficult to use for parameter sensitivity analyses (and also hard to interpret). An approximation for its main (real) branch can be found, but it involves a double logarithm, which still results in divergence of certain analyses (below). Therefore instead, we directly derive an approximation to the solution of the integral above, as follows.

First, we take a log of both sides of the equation:

$$\log(b_{max}) - \alpha(r + z_J) - \log(\kappa + r + z) = 0 \tag{6}$$

As such, this equation still cannot be solved for r_m because of the term $\ln(\kappa + r + z)$. But we can approximate just this term by performing a power series expansion of it around $r_m = 0$:

In [13]:

```python
series(ln(r_m + kappa + z),r_m, 0,3)
```

Out[13]: $\log(\kappa + z) + \frac{r_m}{\kappa + z} - \frac{r_m^2}{2(\kappa + z)^2} + O\left(r_m^3\right)$

That is,

$$\frac{b_{max} e^{-\alpha z_J}}{z e^{\alpha r_m} + (\kappa + r_m) e^{\alpha r_m}} \approx \log(\kappa + z) + \frac{r_m}{\kappa + z} - \frac{r_m^2}{2\kappa^2 + 4\kappa z + 2z^2} + \mathcal{O}\left(r_m^3\right)$$

Then, substituting the first two terms back into the integral solution and solving for r_m gives:

In [14]:

```python
EuLo_int_app = b_max*exp(-alpha*(r_m + z_J))/ exp(log(kappa + z) + r_m/(kappa + z))

r_SP_app = solve(EuLo_int_app-1, r_m); r_SP_app = simplify(r_SP_app[0]); r_SP_app
```

Out[14]: $\frac{-\alpha\kappa z_J - \alpha z z_J + \log\left(b_{max}^{\kappa + z}(\kappa + z)^{-\kappa - z}\right)}{\alpha\kappa + \alpha z + 1}$

Thus the approximation is:

$$r_m \approx \frac{-\alpha\kappa z_J - \alpha z z_J + \kappa \log\left(\frac{b_{max}}{\kappa + z}\right) + z \log\left(\frac{b_{max}}{\kappa + z}\right)}{\alpha(\kappa + z) + 1}$$

that is,

$$r_m \approx \frac{(\kappa + z)\left(\log\left(\frac{b_{max}}{\kappa + z}\right) - \alpha z_J\right)}{\alpha(\kappa + z) + 1}$$

The figures below show that this approximation is very good as long as r_m is relatively small (approx. < 0.5). This range of r_m values is typically where maximal growth rates of arthropods typically lie (Frazier et al 2006).

In [15]:

```python
from scipy.special import lambertw

bmax_vec = np.arange(.1, 1000, 0.1).astype(float) #vector of b_max's

#use lambidy to speed up:
r_SP_app_lam = lambdify((b_max, z_J, z, kappa, alpha), r_SP_app, np)
r_SP_app_vec = r_SP_app_lam(bmax_vec, z_J_par, z_par, kap_par, alp_par)

# Specify r_SP with lambertw:
tmp = lambertw((alp_par*bmax_vec*exp(alp_par*(kap_par + z_par - z_J_par))).astype('float')).real
r_SP_vec = -(kap_par + z_par) + tmp/alp_par

fig = plt.figure(); ax = fig.add_subplot(111)
ax.plot(r_SP_vec, r_SP_app_vec)
ax.plot(np.arange(-1,5,0.1), np.arange(-1,5,0.1), "k--")
ax.set_xlim([min(r_SP_vec),max(r_SP_vec)])
ax.set_ylim([min(r_SP_app_vec),max(r_SP_app_vec)])
ax.set_xlabel('$r$ (exact)', fontsize=14);
ax.set_ylabel('$r$ (approximation)', fontsize=14)

#plt.savefig('../results/Figs/rapprox0.pdf', bbox_inches='tight')
```

Out[15]: `Text(0, 0.5, '$r$ (approximation)')`

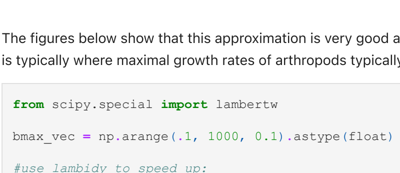

In [16]:

```python
fig = plt.figure(); ax = fig.add_subplot(111)
ax.plot(r_SP_vec, r_SP_vec/r_SP_app_vec)
ax.set_xlabel('Exact $r_m$', fontsize=14)
ax.set_ylabel(r'Approximation ($\%$ of exact $r_m$)', fontsize=14)

#plt.savefig('../results/Figs/rapprox1.pdf', bbox_inches='tight')
```

Out[16]: `Text(0, 0.5, 'Approximation ($\\%$ of exact $r_m$)')`

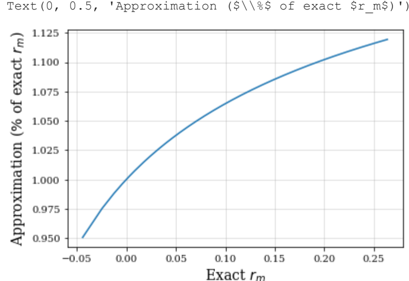

The parameters underlying (r_m) fitness and their units are summarized in the following table:

Parameter	Units	Description
a	day	Age of maturation (egg to adult development time)
b_{max}	individuals (eggs) $\times$ individual (female) $\times$ day $^{-1}$, i.e., day^{-1}	Maximum fecundity (reproductive rate)
z	day^{-1}	Adult mortality rate
z_J	day^{-1}	Mortality rate across all juvenile stages
κ	day^{-1}	Fecundity loss rate

Temperature-dependence (TPCs) of life history traits

We describe the thermal performance curve for each life history parameter using the Sharpe-Schoolfield model, or its inverse:

In [17]:

```python
B_0, E, E_D, T_pk, k, T, T_ref = var('B_0 E E_D T_pk k T T_ref') # Assign symbolic variables
```

In [18]:

```python
B = B_0 * exp(-E * ((1/(k*(T))) - (1/(k*T_ref)))) /(1 + (E / (E_D - E)) * exp((E_D / k)* ((1/T_pk) - (1/T))));
```

Out[18]: $-\frac{B_0 (E - E_D) e^{\frac{E(T - T_{ref})}{T T_{ref} k}}}{E e^{\frac{E_D(T - T_{pk})}{T T_{pk} k}} - E + E_D}$

In [19]:

```python
B_inv = simplify(1/B); B_inv
```

Out[19]: $\frac{\left(-E e^{\frac{E_D(T - T_{pk})}{T T_{pk} k}} + E - E_D\right) e^{-\frac{E(T - T_{ref})}{T T_{ref} k}}}{B_0 (E - E_D)}$

The Sharpe-Schoolfield equation assumes thermodyanmic constraints on a single rate-limiting enzyme governing the metabolic rates underlying the thermal performance curves of life history traits. It therefore provides a mechanistic basis for the hotter-is-better pattern commonly observed in empirical analyses. The parameters of the model are decribed in the main text. One key point to note here is that the constant B_0 implicitly includes the effect of body size scaling. That is, B_0 can actually be written as:

$$B_0 = B_0' m^{\psi},$$

Where m is the body mass of the organism, ψ a scaling exponent determined by fundamental biophysical constraints on individual-level metabolic rate imposed by body size (West et al 1997; Gillooly et al 2002), and B_0' is a size- and temperature-independent constant. This constant can in fact change with thermal adaptation or acclimation. In particular, chronically cooler temperatures are expected to select for an elevation of the B_0' of respiration rate (Angilletta 2009) (and therefore elevate or depress the analogous normalisation constant of different life-history traits - see below), and chronically warmer temperatures may select for a lowering of B_0' of respiration rate. Here we assume that the effects of such changes in B_0' on the temperature-depndence of population fitness, through changes in the B_0 of life history traits, will be small compared to the effect of changes in body mass (scaling) component itself. To account for the latter, we size-correct our empirically estimated values for r_m (see SM). Furthermore, recent work by Kordas et al shows that both E and ψ or respiration rates in aquatic stages of insects too change significantly with chronic expsure to warmer or cooler temperatures. While this will not change our results qualitatively (i.e., it will change the predicted strengths of thermal selection on traits, but not their ranking), this merits further investigation in future work.

TPC parameterizations

We parameterise the Sharpe-Schoolfield equation for the different life-history traits using a set of values lying within empirically feasible ranges (Dell et al 2011) (Table below).

Life history trait	Normalization constant (B_0)	Temperature of peak performance (T_{pk})	Thermal Sensitivity (E)	Life history trait (E_D)
α	25	0-35	0.6	4
b_{max}	1	0-35	0.6	4
z	0.03	0-35	0.6	4
z_J	0.05	0-35	0.6	4
κ	0-1	0-35	0.6	4

Because all five life history traits are ultimately governed by metabolic rate, we assume that they all have the same thermal sensitivity (E) and deactivation energy (E_D), consistent with the median values found in our empirical data (Main text; SM). These values are also broadly consistent with the results of Dell *et al.* 2011. The parameter values for B_0 were varied with type of trait, fixing them to be the median values observed in our empirical dataset (SM). In the absence of appropriate data on the rate of loss of fecundity (κ), we evaluate the model for a range of values (see below). The values of trait T_{pk}'s were varied relative to 25°C, which is the approximate median of the values observed in thge data (SM).

In [20]:

```python
k_par = 8.617 * 10**-5
E_par = .6
E_D_par = 4
T_pk_par = 25
T_ref_par = 10+273.15
B_0_par = 1

T_vec = 273.15+np.arange(0, 40, 0.05) #Vector of temperatures

#Generate some illustrative curves:

B_lam = lambdify((B_0, E, T_pk, T_ref, E_D,k,T), B, np)
B_vec0 = B_lam(B_0_par,E_par,T_pk_par-5+273.15,T_ref_par,E_D_par,k_par,T_vec)
B_vec1 = B_lam(B_0_par,E_par,T_pk_par+273.15,T_ref_par,E_D_par,k_par,T_vec)
B_vec2 = B_lam(B_0_par,E_par,T_pk_par+5+273.15,T_ref_par,E_D_par,k_par,T_vec)

B_inv_lam = lambdify((B_0, E, T_pk, T_ref, E_D,k,T), B_inv, np)
B_inv_vec0 = B_inv_lam(1/B_0_par,E_par,T_pk_par-5+273.15,T_ref_par,E_D_par,k_par,T_vec) #inverse because we are
B_inv_vec1 = B_inv_lam(1/B_0_par,E_par,T_pk_par+273.15,T_ref_par,E_D_par,k_par,T_vec)
B_inv_vec2 = B_inv_lam(1/B_0_par,E_par,T_pk_par+5+273.15,T_ref_par,E_D_par,k_par,T_vec)

#Assign trait-specific TPC values:

B_0_alp =  1/alp_par#days; inverse because we are using inverse of the Sharpe-Schoolfield model
T_pk_alp = T_pk_par
E_alp = E_par
E_D_alp = E_D_par

B_0_bmax = b_max_par #individuals/(individual * day)
T_pk_bmax = T_pk_par
E_bmax = E_par
E_D_bmax = E_D_par

B_0_z = 1/z_par  #1/day; inverse because we are using inverse of the S-S TPC model
T_pk_z = T_pk_par
E_z = E_par
E_D_z = E_D_par

B_0_zJ = 1/z_J_par  #1/day; inverse because we are using inverse of the S-S TPC model
T_pk_zJ = T_pk_par
E_zJ = E_par
E_D_zJ = E_D_par

B_0_kap = kap_par #1/day
T_pk_kap = T_pk_par
E_kap = E_par
E_D_kap = E_D_par

#Calculate the trait TPCs for 3 temperatures

alp_vec0 = B_inv_lam(B_0_alp,E_alp,T_pk_alp-5+273.15,T_ref_par,E_D_alp,k_par,T_vec)
bmax_vec0 = B_lam(B_0_bmax,E_bmax,T_pk_bmax-5+273.15,T_ref_par,E_D_bmax,k_par,T_vec)
z_vec0 = B_inv_lam(B_0_z,E_z,T_pk_z-5+273.15,T_ref_par,E_D_z,k_par,T_vec)
zJ_vec0 = B_inv_lam(B_0_zJ,E_zJ,T_pk_zJ-5+273.15,T_ref_par,E_D_zJ,k_par,T_vec)
kap_vec0 = B_lam(B_0_kap,E_kap,T_pk_kap-5+273.15,T_ref_par,E_D_kap,k_par,T_vec)

alp_vec1 = B_inv_lam(B_0_alp,E_alp,T_pk_alp+273.15,T_ref_par,E_D_alp,k_par,T_vec)
bmax_vec1 = B_lam(B_0_bmax,E_bmax,T_pk_bmax+273.15,T_ref_par,E_D_bmax,k_par,T_vec)
z_vec1 = B_inv_lam(B_0_z,E_z,T_pk_z+273.15,T_ref_par,E_D_z,k_par,T_vec)
zJ_vec1 = B_inv_lam(B_0_zJ,E_zJ,T_pk_zJ+273.15,T_ref_par,E_D_zJ,k_par,T_vec)
kap_vec1 = B_lam(B_0_kap,E_kap,T_pk_kap+273.15,T_ref_par,E_D_kap,k_par,T_vec)

alp_vec2 = B_inv_lam(B_0_alp,E_alp,T_pk_alp+5+273.15,T_ref_par,E_D_alp,k_par,T_vec)
bmax_vec2 = B_lam(B_0_bmax,E_bmax,T_pk_bmax+5+273.15,T_ref_par,E_D_bmax,k_par,T_vec)
z_vec2 = B_inv_lam(B_0_z,E_z,T_pk_z+5+273.15,T_ref_par,E_D_z,k_par,T_vec)
zJ_vec2 = B_inv_lam(B_0_zJ,E_zJ,T_pk_zJ+5+273.15,T_ref_par,E_D_zJ,k_par,T_vec)
kap_vec2 = B_lam(B_0_kap,E_kap,T_pk_kap+5+273.15,T_ref_par,E_D_kap,k_par,T_vec)
```

Temperature Dependence of r_m

To determine the temperature dependence of maximum population growth rate, we can substitute the thermal performance curves of the life history traits into the equation for r_m derived above.

In [21]:

```python
#Calculate r_m
r_m_vec0 = r_SP_app_lam(bmax_vec0, zJ_vec0, z_vec0, kap_vec0, alp_vec0)
r_m_vec1 = r_SP_app_lam(bmax_vec1, zJ_vec1, z_vec1, kap_vec1, alp_vec1)
r_m_vec2 = r_SP_app_lam(bmax_vec2, zJ_vec2, z_vec2, kap_vec2, alp_vec2)
```

Trait sensitivity analysis

We now determine how TPC's of the different traits influence the predicted temperature-dependence of maximum population growth rate (r_m). This will also guide our subsequent calculation of thermal selection gradients for traits. For this sensitivity analysis, we use the chain rule, which allows us to write the thermal sensitivity of r_m in terms of the sums of the product of the partial derivative of r_m with respect to a trait and the derivative of that trait with respect to temperature:

$$\frac{\mathrm{d}r_m}{\mathrm{d}T} = \frac{\partial r_m}{\partial b_{max}}\frac{\mathrm{d}b_{max}}{\mathrm{d}T} + \frac{\partial r_m}{\partial \alpha}\frac{\mathrm{d}\alpha}{\mathrm{d}T} + \frac{\partial r_m}{\partial z}\frac{\mathrm{d}z}{\mathrm{d}T} + \frac{\partial r_m}{\partial z_J}\frac{\mathrm{d}z_J}{\mathrm{d}T} + \frac{\partial r_m}{\partial \kappa}\frac{\mathrm{d}\kappa}{\mathrm{d}T} \tag{7}$$

Each summer term in the RHS of this equation quantifis the relative contribution of the TPC of a parameter/trait to the temperature dependence of r_m.

In [22]:

```python
dr_dbmax = diff(r_SP_app, b_max); simplify(dr_dbmax)
```

Out[22]: $\frac{\kappa + z}{b_{max}(\alpha\kappa + \alpha z + 1)}$

In [23]:

```python
dr_dalp = diff(r_SP_app, alpha); simplify(dr_dalp)
```

Out[23]: $\frac{(\kappa + z)\left(\alpha\kappa z_J + \alpha z z_J - z_J(\alpha\kappa + \alpha z + 1) - \log\left(b_{max}^{\kappa + z}(\kappa + z)^{-\kappa - z}\right)\right)}{(\alpha\kappa + \alpha z + 1)^2}$

In [24]:

```python
dr_dz = diff(r_SP_app, z); simplify(dr_dz)
```

Out[24]: $\frac{-\alpha\kappa - \alpha z - \alpha z_J + \log(b_{max}) - \log(\kappa + z) - 1}{\alpha^2\kappa^2 + 2\alpha^2\kappa z + \alpha^2 z^2 + 2\alpha\kappa + 2\alpha z + 1}$

In [25]:

```python
dr_dzJ = diff(r_SP_app, z_J); simplify(dr_dzJ)
```

Out[25]: $-\frac{\alpha(\kappa + z)}{\alpha\kappa + \alpha z + 1}$

In [26]:

```python
dr_dkap = diff(r_SP_app, kappa); simplify(dr_dkap)
```

Out[26]: $\frac{-\alpha\kappa - \alpha z - \alpha z_J + \log(b_{max}) - \log(\kappa + z) - 1}{\alpha^2\kappa^2 + 2\alpha^2\kappa z + \alpha^2 z^2 + 2\alpha\kappa + 2\alpha z + 1}$

For the TPC model, we ony need to calculate the derivative wrt T for either the function B (for parameters that are rates) or the $1/B$ (for parameters that are times):

In [27]:

```python
dBdT =  diff(B, T); simplify(dBdT)
```

Out[27]: $\frac{B_0 E(E - E_D)\left(E_D e^{\frac{E T_{pk}(T - T_{ref}) + E_D T_{ref}(T - T_{pk})}{T T_{pk} T_{ref} k}} - \left(E e^{\frac{E_D(T - T_{pk})}{T T_{pk} k}} - E + E_D\right) e^{\frac{E(T - T_{ref})}{T T_{ref} k}}\right)}{T^2 k\left(E e^{\frac{E_D(T - T_{pk})}{T T_{pk} k}} - E + E_D\right)^2}$

In [28]:

```python
dB_invdT =  diff(B_inv, T); simplify(dB_invdT)
```

Out[28]: $\frac{E\left(e^{\frac{E_D}{T_{pk}k} - \frac{E_D}{Tk}} - 1\right) e^{-\frac{E}{T_{ref}k} + \frac{E}{Tk}}}{B_0 T^2 k}$

In [29]:

```python
#Get the lambdified pd functions

dr_dalp_lam = lambdify((b_max, alpha, z, z_J, kappa), dr_dalp, np) # The dr_m / dTrait terms
dr_dbmax_lam = lambdify((b_max, alpha, z, kappa), dr_dbmax, np)
dr_dz_lam = lambdify((b_max, alpha, z, z_J, kappa), dr_dz, np)
dr_dzJ_lam = lambdify((alpha, z, kappa), dr_dzJ, np)
dr_dkap_lam = lambdify((b_max, alpha, z, z_J, kappa), dr_dkap, np)

dalp_dT_lam = lambdify((B_0,E,T_pk,T_ref,E_D,k,T), dB_invdT, np) # The dTrait / dT terms
dbmax_dT_lam = lambdify((B_0,E,T_pk,T_ref,E_D,k,T), dBdT, np)
dz_dT_lam =  lambdify((B_0,E,T_pk,T_ref,E_D,k,T), dB_invdT, np)
dzJ_dT_lam =  lambdify((B_0,E,T_pk,T_ref,E_D,k,T), dB_invdT, np)
dkap_dT_lam =  lambdify((B_0,E,T_pk,T_ref,E_D,k,T), dBdT, np)

# Numerically evaluate

dr_dalp_vec = dr_dalp_lam(bmax_vec1, alp_vec1, z_vec1, zJ_vec1, kap_vec1)
dr_dbmax_vec = dr_dbmax_lam(bmax_vec1, alp_vec1, z_vec1, kap_vec1)
dr_dz_vec = dr_dz_lam(bmax_vec1, alp_vec1, z_vec1, zJ_vec1, kap_vec1)
dr_dzJ_vec =  dr_dzJ_lam(alp_vec1, z_vec1, kap_vec1)
dr_dkap_vec = dr_dkap_lam(bmax_vec1, alp_vec1, z_vec1, zJ_vec1, kap_vec1)

dalp_dT_vec = dalp_dT_lam(B_0_alp,E_alp,T_pk_alp+273.15,T_ref_par,E_D_alp,k_par,T_vec)
dbmax_dT_vec = dbmax_dT_lam(B_0_bmax,E_bmax,T_pk_bmax+273.15,T_ref_par,E_D_bmax,k_par,T_vec)
dz_dT_vec =  dz_dT_lam(B_0_z,E_z,T_pk_z+273.15,T_ref_par,E_D_z,k_par,T_vec)
dzJ_dT_vec =  dzJ_dT_lam(B_0_zJ,E_zJ,T_pk_zJ+273.15,T_ref_par,E_D_zJ,k_par,T_vec)
dkap_dT_vec =  dkap_dT_lam(B_0_kap,E_kap,T_pk_kap+273.15,T_ref_par,E_D_kap,k_par,T_vec)

# Calculate the parameter sensitivities

dr_dalp_dT_vec = dr_dalp_vec * dalp_dT_vec
dr_dbmax_dT_vec = dr_dbmax_vec * dbmax_dT_vec
dr_dz_dT_vec = dr_dz_vec * dz_dT_vec
dr_dzJ_dT_vec = dr_dzJ_vec * dzJ_dT_vec
dr_dkap_dT_vec = dr_dkap_vec * dkap_dT_vec

dr_dT_vec = dr_dbmax_dT_vec + dr_dalp_dT_vec + dr_dz_dT_vec + dr_dzJ_dT_vec + dr_dkap_dT_vec
```

In [30]:

```python
######## Fig 1 ############
fig = plt.figure(figsize=(10, 8));
cm = plt.cm.copper_r(np.linspace(0, 1, 3))
csfont = {'fontname':'DejaVu Sans'}

####### Plot r_m TPCs######

ax = fig.add_subplot(221)

ax.set_prop_cycle('color',cm)

ax.plot(T_vec-273.15, r_m_vec0, linewidth=2.5)
ax.plot(T_vec-273.15, r_m_vec1, linewidth=2.5)
ax.plot(T_vec-273.15, r_m_vec2, linewidth=2.5)

ax.plot([T_pk_par, T_pk_par], [-.08,max(r_m_vec1)], color='k', linestyle='--', linewidth=1)
ax.plot([0,T_pk_par], [max(r_m_vec1), max(r_m_vec1)], color='k', linestyle='--', linewidth=1)
ax.text(0+.5, max(r_m_vec1), '$r_{opt}$',  horizontalalignment='left', verticalalignment='bottom', fontsize=13)

ax.set_title('A', weight="bold", fontsize=14, x=.05, y=.9,**csfont);
ax.set_xlim(0,max(T_vec)-273.15); ax.set_ylim(-.08,max(r_m_vec2)*(1+0.05));
ax.text(T_pk_par, ax.get_ylim()[0], '$T_{opt}$',  horizontalalignment='right', verticalalignment='bottom', font
# ax.plot(T_vec-273.15, [0]*len(T_vec), color='k', linestyle='-.', linewidth=1);
ax.set_xlabel(r'Temperature  ($^\circ$C)', fontsize=13);
ax.set_ylabel('Maximum population\n growth rate ($r_m$)', fontsize=13)
ax.tick_params(axis='x', labelsize=13);
ax.tick_params(axis='y', labelsize=13);
ax.axes.xaxis.set_ticklabels([])

####### Plot results of sensitivity analysis######

ax = fig.add_subplot(223)
ax.set_title('C', weight="bold", fontsize=14, x=.05, y=.9,**csfont);

ax.plot(T_vec-273.15,dr_dT_vec, color=cm[1],linewidth=3.5, linestyle='-')
ax.plot(T_vec-273.15,dr_dalp_dT_vec, color=cm[1],linewidth=3, linestyle='--')
ax.plot(T_vec-273.15,dr_dbmax_dT_vec, color=cm[1],linewidth=2.5, linestyle='--')
ax.plot(T_vec-273.15,dr_dzJ_dT_vec, color=cm[1],linewidth=2, linestyle='--')
ax.plot(T_vec-273.15,dr_dz_dT_vec, color=cm[1],linewidth=1.5, linestyle='--')
ax.plot(T_vec-273.15,dr_dkap_dT_vec, color=cm[1],linewidth=1, linestyle='--')
ax.plot([T_pk_par, T_pk_par], [ax.get_ylim()[0],ax.get_ylim()[1]], color='k', linestyle='--', linewidth=1)
ax.set_xlim(0,max(T_vec)-273.15); ax.set_ylim(-0.023,0.017)
ax.text(T_pk_par, ax.get_ylim()[0], '$T_{opt}$',  horizontalalignment='right', verticalalignment='bottom', font
ax.set_xlabel(r'Temperature  ($^\circ$C)', fontsize=14);
ax.set_ylabel('Derivative of\n temperature-dependence', fontsize=14);
ax.tick_params(axis='x', labelsize=13);
ax.tick_params(axis='y', labelsize=13);
plt.legend([r'$r_m$', r'$\alpha$', '$b_{max}$', '$z_J$', '$z$', r'$\kappa$'], loc='lower left',fontsize=11)

####### Plot illustrative SS TPCs######

ax = fig.add_subplot(222)
ax.set_prop_cycle('color',cm)
ax.plot(T_vec-273.15, B_vec0, linewidth=2)
ax.plot(T_vec-273.15, B_vec1, linewidth=2)
ax.plot(T_vec-273.15, B_vec2, linewidth=2)

E_arrow_ran = [15,20]; E_arrow_disp = 1.08
ax.annotate('', xy=(E_arrow_ran[0], B_vec1[np.where(T_vec-273.15 == E_arrow_ran[0])[0][0]]*E_arrow_disp),
            xytext=(E_arrow_ran[1],B_vec1[np.where(T_vec-273.15 == E_arrow_ran[1])[0][0]]*E_arrow_disp),
            arrowprops=dict(arrowstyle='<-'), horizontalalignment='left',verticalalignment='top')
ax.text(np.mean(E_arrow_ran),B_vec1[np.where(T_vec-273.15 == np.mean(E_arrow_ran))[0][0]]*E_arrow_disp+.25, '$E
       horizontalalignment='right', verticalalignment='top', fontsize=13)

E_D_arrow_ran = [29,31];E_D_arrow_disp = 1.12
ax.annotate('', xy=(E_D_arrow_ran[0],B_vec1[np.where(T_vec-273.15 == E_D_arrow_ran[0])[0][0]]*E_D_arrow_disp),
            xytext=(E_D_arrow_ran[1],B_vec1[np.where(T_vec-273.15 == E_D_arrow_ran[1])[0][0]]*E_D_arrow_disp),
            arrowprops=dict(arrowstyle='<-'), horizontalalignment='left',verticalalignment='top')
ax.text(np.mean(E_D_arrow_ran),B_vec1[np.where(T_vec-273.15 == np.mean(E_D_arrow_ran))[0][0]]*E_D_arrow_disp+.[illegible]
       horizontalalignment='left', verticalalignment='top', fontsize=13)

ax.plot([T_pk_par, T_pk_par], [0,max(B_vec1)], color='k', linestyle='--', linewidth=1)
ax.text(T_pk_par, 0, '$T_{pk}$',  horizontalalignment='left', verticalalignment='bottom', fontsize=13)

ax.plot([0,T_pk_par], [max(B_vec1), max(B_vec1)], color='k', linestyle='--', linewidth=1)
ax.text(0, max(B_vec1), '$B_{pk}$',  horizontalalignment='left', verticalalignment='bottom', fontsize=13)

ax.plot([0,10], [B_0_par, B_0_par], color='k', linestyle='--', linewidth=1)
ax.plot([10, 10], [0, B_0_par], color='k', linestyle='--', linewidth=1)
ax.text(0, B_0_par, '$B_0$',  horizontalalignment='left', verticalalignment='bottom', fontsize=13)
ax.text(10, 0, ' $T_{ref}$',  horizontalalignment='left', verticalalignment='bottom', fontsize=13)

ax.plot(T_pk_par-5, max(B_vec0), marker='^', markersize=8, color="red",alpha = 0.7)
ax.plot(T_pk_par, max(B_vec1), marker='^', markersize=8, color="red",alpha = 0.7)
ax.plot(T_pk_par+5, max(B_vec2), marker='^', markersize=8, color="red",alpha = 0.7)

ax.set_title('B', weight="bold", fontsize=14, x=.05, y=.9,**csfont);
ax.set_ylabel('Trait Value ($B$)', fontsize=14)
ax.tick_params(axis='x', labelsize=13);
ax.axes.get_yaxis().set_ticks([]) # because values are arbitrary
ax.set_xlim(0,max(T_vec)-273.15);
ax.set_ylim(0,)
ax.axes.xaxis.set_ticklabels([])

####### Plot illustrative inverse SS TPCs#######

ax = fig.add_subplot(224)
ax.set_prop_cycle('color',cm)
ax.plot(T_vec-273.15, B_inv_vec0, linewidth=2)
ax.plot(T_vec-273.15, B_inv_vec1, linewidth=2)
ax.plot(T_vec-273.15, B_inv_vec2, linewidth=2)

ax.plot(T_pk_par-5, min(B_inv_vec0), marker='o', markersize=8, color="blue",alpha = 0.7)
ax.plot(T_pk_par, min(B_inv_vec1), marker='o', markersize=8, color="blue",alpha = 0.7)
ax.plot(T_pk_par+5, min(B_inv_vec2), marker='o', markersize=8, color="blue",alpha = 0.7)

ax.tick_params(axis='x', labelsize=13);
ax.set_ylabel('Trait Value ($B$)', fontsize=14)
ax.axes.get_yaxis().set_ticks([]) # because values are arbitrary
ax.set_xlim(0,max(T_vec)-273.15);
ax.set_ylim(0,2.5);
ax.axes.get_yaxis().set_ticks([])
ax.set_xlabel(r'Temperature  ($^\circ$C)', fontsize=14);

plt.subplots_adjust(wspace = 0.15,hspace=.01)

# plt.savefig('../results/Figs/r_TPC.pdf', bbox_inches='tight')
```

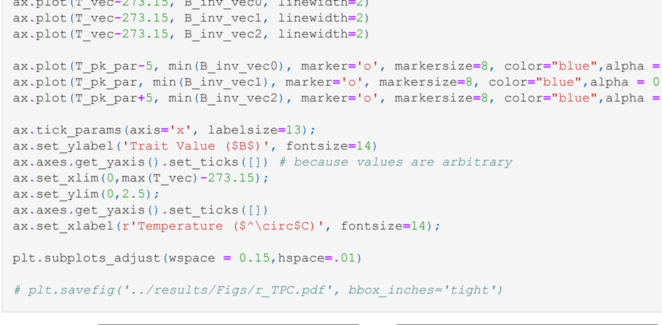

In **C**, the relative contribution of each trait's TPC to the temperature dependence of r_m (i.e., $\frac{dr_m}{dT}$ - the black dash-dotted line) is the deviation of its (partial) derivative line from the $\frac{dr_m}{dT}$ line; greater the deviation, greater its contribution (and therefore sensitivity of $\frac{dr_m}{dT}$ to it).

We can perform another type of trait sensitivity analysis (holding one trait to be temperature-independent at a time):

In [31]:

```python
r_alp_cons_vec = r_SP_app_lam(bmax_vec1,zJ_vec1, z_vec1, kap_vec1, alp_par)
r_bmax_cons_vec = r_SP_app_lam(b_max_par,zJ_vec1, z_vec1, kap_vec1, alp_vec1)
r_z_cons_vec = r_SP_app_lam(bmax_vec1,zJ_vec1, z_par, kap_vec1, alp_vec1)
r_zJ_cons_vec = r_SP_app_lam(bmax_vec1,z_J_par, z_vec1, kap_vec1, alp_vec1)
r_kap_cons_vec = r_SP_app_lam(bmax_vec1,zJ_vec1, z_vec1, kap_par, alp_vec1)

############# Additional sesnitivity analysis figure ################

fig = plt.figure(figsize=(5, 4))
ax = fig.add_subplot()
# ax.set_title('B', weight="bold", fontsize=13, x=.05, y=.9,**csfont);
ax.plot(T_vec-273.15,r_m_vec1, 'black',linewidth=3, linestyle='-.');
ax.plot(T_vec-273.15,r_alp_cons_vec, 'blue');
ax.plot(T_vec-273.15,r_bmax_cons_vec, 'red');
ax.plot(T_vec-273.15,r_zJ_cons_vec, 'orange');
ax.plot(T_vec-273.15,r_z_cons_vec, 'cyan');
ax.plot(T_vec-273.15,r_kap_cons_vec, 'purple');
ax.plot([T_pk_par, T_pk_par], [ax.get_ylim()[0],ax.get_ylim()[1]], color='k', linestyle='--', linewidth=1)
ax.set_xlim(0,36)
ax.set_ylim(-0.1,.2)
ax.set_xlabel(r'Temperature  ($^\circ$C)', fontsize=14);
ax.set_ylabel('$r_m$', fontsize=14);
plt.legend(['Full', r'$\alpha$  const', '$b_{max}$ const', '$z_J$ const', '$z$  const', r'$\kappa$ const'], loc[illegible]

plt.subplots_adjust(wspace=0.3)

#plt.savefig('../results/Figs/r_sens.pdf', bbox_inches='tight')
```

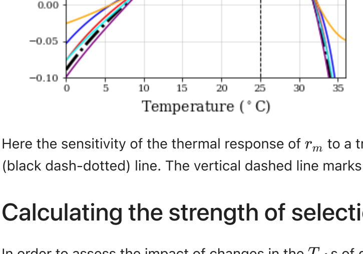

Here the sensitivity of the thermal response of r_m to a trait's TPC is the deviation of the line of that trait *away* from the full model (black dash-dotted) line. The vertical dashed line marks the optimal temperature (T_{opt}) for r_m }.

Calculating the strength of selection on traits

In order to assess the impact of changes in the T_{pk}s of different traits and lifestages on population fitness, we re-calculate the temperature-dependence of r_m (its TPC) with respect to changes in each of them, holding all others constant. We do not include κ as a focal trait for this because it has the weakest influence on r_m (previous section), and also because there are almost no data on its temperature dependence.

In [32]:
```python
T_vec = 273.15+np.arange(0, 40, 0.05) #Vector of temperatures

alp_vec = B_inv_lam(B_0_alp,E_alp,T_pk_alp+273.15,T_ref_par,E_D_alp,k_par,T_vec)
bmax_vec = B_lam(B_0_bmax,E_bmax,T_pk_bmax+273.15,T_ref_par,E_D_bmax,k_par,T_vec)
z_vec = B_inv_lam(B_0_z,E_z,T_pk_z+273.15,T_ref_par,E_D_z,k_par,T_vec)
zJ_vec = B_inv_lam(B_0_zJ,E_zJ,T_pk_zJ+273.15,T_ref_par,E_D_zJ,k_par,T_vec)
kap_vec = B_lam(B_0_kap,E_kap,T_pk_kap+273.15,T_ref_par,E_D_kap,k_par,T_vec)

r_m_vec = r_SP_app_lam(bmax_vec, zJ_vec, z_vec, kap_vec, alp_vec)

T_pk_mm = T_pk_par + np.arange(-10,15.5,0.5) # new peaks
T_ref_mm = T_ref_par
```

In [33]:
```python
############# Fig. 2 ###############
fntsiz = 17
fig = plt.figure(figsize=(19, 8))

#========== for alpha ==================
r_opt_vec = ["NA"]*len(T_pk_mm)

ax = fig.add_subplot(241)
ax.set_title('Development time '+r'($\alpha$)', fontsize=fntsiz, **csfont)
ax.text(0.02, 0.98, 'A',
        horizontalalignment='left', verticalalignment='top', **csfont,
        fontsize=fntsiz, weight="bold", transform=ax.transAxes)
colormap = plt.cm.copper_r
cm = colormap(np.linspace(0, 1, len(T_pk_mm)))
ax.set_prop_cycle('color',cm)

for i in range(len(T_pk_mm)):

    alp_mm_tmp_vec = B_inv_lam(B_0_alp,E_alp,T_pk_mm[i]+273.15,T_ref_mm,E_D_alp,k_par,T_vec)
    r_mm_tmp_vec = r_SP_app_lam(bmax_vec, zJ_vec, z_vec, kap_vec, alp_mm_tmp_vec)
    r_opt_vec[i] = max(r_mm_tmp_vec)
    ax.plot(T_vec-273.15, r_mm_tmp_vec, label = r'$\Delta T_{pk} = $ ' + str(i))
    if i == min(range(len(T_pk_mm))):
        tmp_x1 = (T_vec-273.15)[np.where(r_mm_tmp_vec == max(r_mm_tmp_vec))[0][0]]
        tmp_y1 = max(r_mm_tmp_vec)
        ax.annotate('', xy=(tmp_x1,tmp_y1),
                    xytext=(tmp_x1-3.5,tmp_y1), arrowprops=dict(arrowstyle='->'))
        ax.text(tmp_x1-3.5,tmp_y1, r'$\Delta T_{pk}$='+"{:.0f}".format(T_pk_mm[0] - T_pk_par)+r'$^\circ$C',
                horizontalalignment='right', verticalalignment='center', fontsize=fntsiz-4)
    elif i == max(range(len(T_pk_mm))) :
        tmp_x1 = (T_vec-273.15)[np.where(r_mm_tmp_vec == max(r_mm_tmp_vec))[0][0]]
        tmp_y1 = max(r_mm_tmp_vec)
        ax.annotate('', xy=(tmp_x1,tmp_y1),
                     xytext=(tmp_x1-3.5,tmp_y1), arrowprops=dict(arrowstyle='->'))
        ax.text(tmp_x1-3.5,tmp_y1, r'$\Delta T_{pk}$=+'+"{:.0f}".format(T_pk_mm[len(T_pk_mm)-1] - T_pk_par)+r'$
                horizontalalignment='right', verticalalignment='center', fontsize=fntsiz-4)

ax.plot(T_vec-273.15, r_m_vec, 'blue', linestyle='-', linewidth = 3)
ax.plot(T_vec-273.15, [0]*len(T_vec), "k--") # r_m = 0 line
ax.set_xlabel(r'Temperature ($^\circ$C)', fontsize=fntsiz)
ax.set_ylabel('Maximum population\n growth rate ($r_m$)', fontsize=fntsiz)
ax.set_ylim(-0.05,0.24)
ax.set_xlim(0,36)
ax.tick_params(axis='x', labelsize=fntsiz); ax.tick_params(axis='y', labelsize=fntsiz)

ax = fig.add_subplot(245) # plot selection gradient
ax.text(0.02, 0.98, 'E',
        horizontalalignment='left', verticalalignment='top', **csfont,
        fontsize=fntsiz, weight="bold", transform=ax.transAxes)
ax.plot(T_pk_mm, r_opt_vec, 'black')
ax.plot(T_pk_mm, [max(r_opt_vec)]*len(T_pk_mm), "k--") # max(r_m,opt) line
ax.set_ylim(0.06,.24)
ax.text(max(T_pk_mm),max(r_opt_vec), '$r_{opt}=$'+"{:.3f}".format(max(r_opt_vec)),
                horizontalalignment='right', verticalalignment='bottom', fontsize=fntsiz-4)

# ax.text(0.5,0.5, '$T_{pk}$ where' + r'$\frac{\partial^2 r_{opt}}{\partial {T_{pk}^\theta}^2}$'+'\nis minimum
        # "{:.0f}".format(T_pk_mm[np.where(np.gradient(np.gradient(r_opt_vec)) == min(np.gradient(np.gradient(r
        #          horizontalalignment='left', verticalalignment='top', fontsize=fntsiz-4, transform=ax.transAxe

tmp_x = T_pk_mm[np.where(np.gradient(np.gradient(r_opt_vec)) == min(np.gradient(np.gradient(r_opt_vec))))][0];
ax.annotate("", xy=(tmp_x, ax.get_ylim()[0]), xytext=(tmp_x, ax.get_ylim()[0]+tmp_y_disp),
            arrowprops=dict(arrowstyle="->"))
ax.text(tmp_x, ax.get_ylim()[0]+tmp_y_disp, "{:.0f}".format(tmp_x)+r'$^\circ$C',
                horizontalalignment='center', verticalalignment='bottom', fontsize=fntsiz-4) # where gradient 

ax.plot([T_pk_par, T_pk_par], [ax.get_ylim()[0],ax.get_ylim()[1]], color='b', linestyle='-', linewidth=1.5)
ax.set_xlabel(r'$T_{pk}^\alpha$ ($^\circ$C)', fontsize=fntsiz)
ax.set_ylabel('Maximum population\n growth rate at $T_{opt}$ ($r_{opt}$)', fontsize=fntsiz)
ax.tick_params(axis='x', labelsize=fntsiz); ax.tick_params(axis='y', labelsize=fntsiz)

# ========= For b_max =====================

r_opt_vec = ["NA"]*len(T_pk_mm)

ax = fig.add_subplot(242)
ax.set_title('Maximum fecundity'+ r' ($b_{max}$)', fontsize=fntsiz, **csfont)
ax.text(0.02, 0.98, 'B',
        horizontalalignment='left', verticalalignment='top', **csfont,
        fontsize=fntsiz, weight="bold", transform=ax.transAxes)
ax.set_prop_cycle('color',cm)

for i in range(len(T_pk_mm)):

    bmax_mm_tmp_vec = B_lam(B_0_bmax,E_bmax,T_pk_mm[i]+273.15,T_ref_mm,E_D_bmax,k_par,T_vec)
    r_mm_tmp_vec = r_SP_app_lam(bmax_mm_tmp_vec, zJ_vec, z_vec, kap_vec, alp_vec)
    r_opt_vec[i] = max(r_mm_tmp_vec)
    ax.plot(T_vec-273.15, r_mm_tmp_vec, label = r'$\Delta T_{pk} = $ ' + str(i))

ax.plot(T_vec-273.15, r_m_vec, 'blue', linestyle='-', linewidth = 3)
ax.plot(T_vec-273.15, [0]*len(T_vec), "k--")
ax.set_xlabel(r'Temperature ($^\circ$C)', fontsize=fntsiz)
ax.set_ylim(-0.05,0.24)
ax.set_xlim(0,36)
ax.tick_params(axis='x', labelsize=fntsiz); ax.tick_params(axis='y', labelsize=fntsiz)

ax = fig.add_subplot(246)# plot selection gradient
ax.text(0.02, 0.98, 'F',
        horizontalalignment='left', verticalalignment='top', **csfont,
        fontsize=fntsiz, weight="bold", transform=ax.transAxes)
ax.plot(T_pk_mm, r_opt_vec, 'black')
ax.plot(T_pk_mm, [max(r_opt_vec)]*len(T_pk_mm), "k--") # max(r_m,opt) line
ax.set_ylim(0.06,.24)
ax.plot([T_pk_par, T_pk_par], [ax.get_ylim()[0],ax.get_ylim()[1]], color='b', linestyle='-', linewidth=1.5)
ax.text(max(T_pk_mm),max(r_opt_vec), '$r_{opt}=$'+"{:.3f}".format(max(r_opt_vec)),
                horizontalalignment='right', verticalalignment='bottom', fontsize=fntsiz-4)
# ax.text(0.5,0.5, '$T_{pk}$ where' + r'$\frac{\partial^2 r_{opt}}{\partial {T_{pk}^\theta}^2}$'+'\nis minimum
#         "{:.0f}".format(T_pk_mm[np.where(np.gradient(np.gradient(r_opt_vec)) == min(np.gradient(np.gradient(r
#                  horizontalalignment='left', verticalalignment='top', fontsize=fntsiz-4, transform=ax.transAxe
tmp_x = T_pk_mm[np.where(np.gradient(np.gradient(r_opt_vec)) == min(np.gradient(np.gradient(r_opt_vec))))][0];
ax.annotate("", xy=(tmp_x, ax.get_ylim()[0]), xytext=(tmp_x, ax.get_ylim()[0]+tmp_y_disp),
            arrowprops=dict(arrowstyle="->"))
ax.text(tmp_x, ax.get_ylim()[0]+tmp_y_disp, "{:.0f}".format(tmp_x)+r'$^\circ$C',
                horizontalalignment='center', verticalalignment='bottom', fontsize=fntsiz-4) # where gradient 

ax.set_xlabel(r'$T_{pk}^{b_{max}}$($^\circ$C)', fontsize=fntsiz)
ax.tick_params(axis='x', labelsize=fntsiz); ax.tick_params(axis='y', labelsize=fntsiz)

# ========= For z_J =====================

r_opt_vec = ["NA"]*len(T_pk_mm)

ax = fig.add_subplot(2,4,3)
ax.set_title('Juvenile mortality rate'+ r' ($z_J$)', fontsize=fntsiz, **csfont)
ax.text(0.02, 0.98, 'C',
        horizontalalignment='left', verticalalignment='top', **csfont,
        fontsize=fntsiz, weight="bold", transform=ax.transAxes)
ax.set_prop_cycle('color',cm)

for i in range(len(T_pk_mm)):

    zJ_mm_tmp_vec = B_inv_lam(B_0_zJ,E_zJ,T_pk_mm[i]+273.15,T_ref_mm,E_D_zJ,k_par,T_vec)
    r_mm_tmp_vec = r_SP_app_lam(bmax_vec, zJ_mm_tmp_vec, z_vec, kap_vec, alp_vec)
    r_opt_vec[i] = max(r_mm_tmp_vec)
    ax.plot(T_vec-273.15, r_mm_tmp_vec, label = r'$\Delta T_{pk} = $ ' + str(i))

ax.plot(T_vec-273.15, r_m_vec, 'blue', linestyle='-', linewidth = 3)
ax.plot(T_vec-273.15, [0]*len(T_vec), "k--")
ax.set_xlabel(r'Temperature ($^\circ$C)', fontsize=fntsiz)
ax.set_ylim(-0.05,0.24)
ax.set_xlim(0,36)
ax.tick_params(axis='x', labelsize=fntsiz); ax.tick_params(axis='y', labelsize=fntsiz)

ax = fig.add_subplot(2,4,7)# plot selection gradient
ax.text(0.02, 0.98, 'G',
        horizontalalignment='left', verticalalignment='top', **csfont,
        fontsize=fntsiz, weight="bold", transform=ax.transAxes)
ax.plot(T_pk_mm, r_opt_vec, 'black')
ax.plot(T_pk_mm, [max(r_opt_vec)]*len(T_pk_mm), "k--") # max(r_m,opt) line
ax.set_ylim(0.06,.24)
ax.plot([T_pk_par, T_pk_par], [ax.get_ylim()[0],ax.get_ylim()[1]], color='b', linestyle='-', linewidth=1.5)
ax.text(max(T_pk_mm),max(r_opt_vec), '$r_{opt}=$'+"{:.3f}".format(max(r_opt_vec)),
                horizontalalignment='right', verticalalignment='bottom', fontsize=fntsiz-4)
# ax.text(0.5,0.5, '$T_{pk}$ where' + r'$\frac{\partial^2 r_{opt}}{\partial {T_{pk}^\theta}^2}$'+'\nis minimum
#         "{:.0f}".format(T_pk_mm[np.where(np.gradient(np.gradient(r_opt_vec)) == min(np.gradient(np.gradient(r
#                  horizontalalignment='left', verticalalignment='top', fontsize=fntsiz-4, transform=ax.transAxe
tmp_x = T_pk_mm[np.where(np.gradient(np.gradient(r_opt_vec)) == min(np.gradient(np.gradient(r_opt_vec))))][0];
ax.annotate("", xy=(tmp_x, ax.get_ylim()[0]), xytext=(tmp_x, ax.get_ylim()[0]+tmp_y_disp),
            arrowprops=dict(arrowstyle="->"))
ax.text(tmp_x, ax.get_ylim()[0]+tmp_y_disp, "{:.0f}".format(tmp_x)+r'$^\circ$C',
                horizontalalignment='center', verticalalignment='bottom', fontsize=fntsiz-4) # where gradient 

ax.set_xlabel(r'$T_{pk}^{z_J}$($^\circ$C)', fontsize=fntsiz)
ax.tick_params(axis='x', labelsize=fntsiz); ax.tick_params(axis='y', labelsize=fntsiz)

# ========= For z =====================

r_opt_vec = ["NA"]*len(T_pk_mm)

ax = fig.add_subplot(2,4,4)
ax.set_title('Adult mortality rate'+ r' ($z$)', fontsize=fntsiz, **csfont)
ax.text(0.02, 0.98, 'D',
        horizontalalignment='left', verticalalignment='top', **csfont,
        fontsize=fntsiz, weight="bold", transform=ax.transAxes)

ax.set_prop_cycle('color',cm)

for i in range(len(T_pk_mm)):
    z_mm_tmp_vec  = B_inv_lam(B_0_z,E_z,T_pk_mm[i]+273.15,T_ref_mm,E_D_z,k_par,T_vec)
    r_mm_tmp_vec = r_SP_app_lam(bmax_vec, zJ_vec, z_mm_tmp_vec, kap_vec, alp_vec)
    r_opt_vec[i] = max(r_mm_tmp_vec)
    ax.plot(T_vec-273.15, r_mm_tmp_vec, label = r'$\Delta T_{pk} = $ ' + str(i))

ax.plot(T_vec-273.15, r_m_vec, 'blue', linestyle='-', linewidth = 3)
ax.plot(T_vec-273.15, [0]*len(T_vec), "k--")
ax.set_xlabel(r'Temperature ($^\circ$C)', fontsize=fntsiz)
ax.set_ylim(-0.05,0.24)
ax.set_xlim(0,36)
ax.tick_params(axis='x', labelsize=fntsiz); ax.tick_params(axis='y', labelsize=fntsiz)

ax = fig.add_subplot(2,4,8)# plot selection gradient
ax.text(0.02, 0.98, 'H',
        horizontalalignment='left', verticalalignment='top', **csfont,
        fontsize=fntsiz, weight="bold", transform=ax.transAxes)
ax.plot(T_pk_mm, r_opt_vec, 'black')
ax.plot(T_pk_mm, [max(r_opt_vec)]*len(T_pk_mm), "k--") # max(r_m,opt) line
ax.set_ylim(0.06,.24)
ax.plot([T_pk_par, T_pk_par], [ax.get_ylim()[0],ax.get_ylim()[1]], color='b', linestyle='-', linewidth=1.5)
ax.text(max(T_pk_mm),max(r_opt_vec), '$r_{opt}=$'+"{:.3f}".format(max(r_opt_vec)),
                horizontalalignment='right', verticalalignment='bottom', fontsize=fntsiz-4)
# ax.text(0.5,0.5, '$T_{pk}$ where' + r'$\frac{\partial^2 r_{opt}}{\partial {T_{pk}^\theta}^2}$'+'\nis minimum
#         "{:.0f}".format(T_pk_mm[np.where(np.gradient(np.gradient(r_opt_vec)) == min(np.gradient(np.gradient(r
#                  horizontalalignment='left', verticalalignment='top', fontsize=fntsiz-4, transform=ax.transAxe
tmp_x = T_pk_mm[np.where(np.gradient(np.gradient(r_opt_vec)) == min(np.gradient(np.gradient(r_opt_vec))))][0];
ax.annotate("", xy=(tmp_x, ax.get_ylim()[0]), xytext=(tmp_x, ax.get_ylim()[0]+tmp_y_disp),
            arrowprops=dict(arrowstyle="->"))
ax.text(tmp_x, ax.get_ylim()[0]+tmp_y_disp, "{:.0f}".format(tmp_x)+r'$^\circ$C',
                horizontalalignment='center', verticalalignment='bottom', fontsize=fntsiz-4) # where gradient 

ax.set_xlabel(r'$T_{pk}^{z}$($^\circ$C)', fontsize=fntsiz)
ax.tick_params(axis='x', labelsize=fntsiz); ax.tick_params(axis='y', labelsize=fntsiz)

fig.tight_layout()

plt.savefig('../results/ThermMM.pdf', bbox_inches='tight')
```

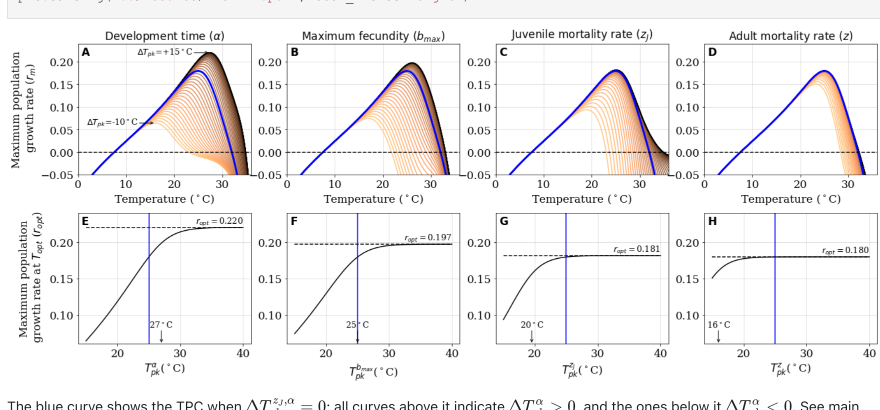

The blue curve shows the TPC when $\Delta T_{pk}^{z_J,\alpha} = 0$; all curves above it indicate $\Delta T_{pk}^{\alpha} > 0$, and the ones below it $\Delta T_{pk}^{\alpha} < 0$. See main text for further explanation.

The effect of physiological mismatches on population fitness

Given that a higher $T_{pk,\alpha}$ increases fitness, and that all trait-specific selection gradients are monotonically increasing, closer the T_{pk}'s of the other traits are to it, higher the $r_{m,opt}$. This can either be measured by the sum of all T_{pk}'s, or their variance. To illustrate this, we will use parameter values for E from the real data.

First load the results of the TPC fitting:

In [34]:
```python
import pandas as pd

alpha_fits =  pd.read_csv('../data/alpha_Tpks_AllParams.csv')
zj_fits =  pd.read_csv('../data/zj_Tpks_AllParams.csv')
z_fits = pd.read_csv('../data/z_Tpks_AllParams.csv')
bmax_fits =  pd.read_csv('../data/bmax_Tpks_AllParams.csv')

alpha_fits.head(10) #Each fit has ten rows of parameter estimates per species
```

Out[34]:

	Unnamed: 0	param	conf_lower	conf_upper	method	estimate	species	trait
0	1	rmax	0.114850	0.177890	residual bootstrap	0.130927	Aedes albopictus	juvenile development rate
1	2	topt	29.873741	33.560590	residual bootstrap	31.160000	Aedes albopictus	juvenile development rate
2	3	ctmin	-2.734000	9.364437	residual bootstrap	4.284000	Aedes albopictus	juvenile development rate
3	4	ctmax	35.997099	45.052775	residual bootstrap	41.121000	Aedes albopictus	juvenile development rate
4	5	thermal_safety_margin	2.607346	14.821406	residual bootstrap	9.961000	Aedes albopictus	juvenile development rate
5	6	thermal_tolerance	30.972476	46.430000	residual bootstrap	36.837000	Aedes albopictus	juvenile development rate
6	7	breadth	3.853889	8.084656	residual bootstrap	6.226000	Aedes albopictus	juvenile development rate
7	8	r_tref	0.018167	0.037033	residual bootstrap	0.025150	Aedes albopictus	juvenile development rate
8	9	e	0.620453	1.136747	residual bootstrap	0.865978	Aedes albopictus	juvenile development rate
9	10	eh	3.345901	20.000000	residual bootstrap	4.741471	Aedes albopictus	juvenile development rate

In [35]:
```python
fig = plt.figure(figsize=(10, 4))
fig.suptitle(r'Development rate ($\alpha$)', fontsize=16)

ax = fig.add_subplot(121)
ax.set_title('A', weight="bold", fontsize=14, x=.03, y=.9,**csfont)
ax.hist(alpha_fits[alpha_fits["param"] == "e"]["estimate"])
ax.set_xlabel(r"Activation Energy ($E$, eV)", fontsize=14)
ax.set_ylabel('Number of species', fontsize=14)
ax.tick_params(axis='x', labelsize=13); ax.tick_params(axis='y', labelsize=13)
ax.text(0.5, 0.8, 'Median = {:2.2f} eV'.format(np.median(alpha_fits[alpha_fits["param"] == "e"]["estimate"])),
        horizontalalignment='left', verticalalignment='top', **csfont,
        fontsize=.8*fntsiz, transform=ax.transAxes)

ax = fig.add_subplot(122)
ax.set_title('B', weight="bold", fontsize=14, x=.03, y=.9,**csfont)
ax.hist(alpha_fits[alpha_fits["param"] == "topt"]["estimate"])
ax.set_xlabel(r"Temperature of peak performance ($T_{pk}$)", fontsize=14)
# ax.set_ylabel('Number of species', fontsize=14)
ax.tick_params(axis='x', labelsize=13); ax.tick_params(axis='y', labelsize=13)

# plt.subplots_adjust(wspace=0.2, hspace=.2)

#plt.savefig('../results/Figs/TPC_data_dists_alpha.pdf', bbox_inches='tight')
```

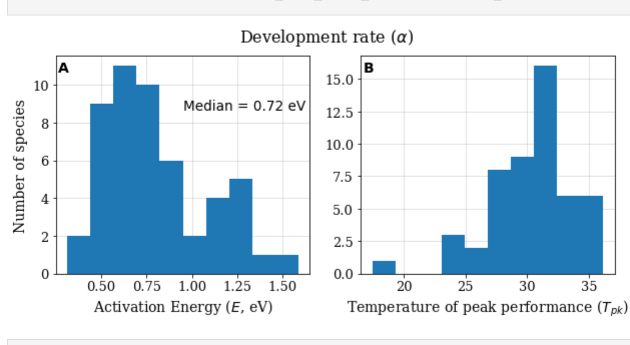

In [36]:
```python
fig = plt.figure(figsize=(10, 4))
fig.suptitle(r'Peak fecundity rate ($b_{max}$)', fontsize=16)

ax = fig.add_subplot(121)
ax.set_title('A', weight="bold", fontsize=14, x=.03, y=.9,**csfont)
ax.hist(bmax_fits[bmax_fits["param"] == "e"]["estimate"])
ax.set_xlabel(r"Activation Energy ($E$, eV)", fontsize=14)
ax.set_ylabel('Number of species', fontsize=14)
ax.tick_params(axis='x', labelsize=13); ax.tick_params(axis='y', labelsize=13)
ax.text(0.5, 0.8, 'Median = {:2.2f} eV'.format(np.median(bmax_fits[bmax_fits["param"] == "e"]["estimate"])),
        horizontalalignment='left', verticalalignment='top', **csfont,
        fontsize=.8*fntsiz, transform=ax.transAxes)

ax = fig.add_subplot(122)
ax.set_title('B', weight="bold", fontsize=14, x=.03, y=.9,**csfont)
ax.hist(bmax_fits[bmax_fits["param"] == "topt"]["estimate"])
ax.set_xlabel(r"Temperature of peak performance ($T_{pk}$)", fontsize=14)
# ax.set_ylabel('Number of species', fontsize=14)
ax.tick_params(axis='x', labelsize=13); ax.tick_params(axis='y', labelsize=13)

plt.subplots_adjust(wspace=0.2, hspace=.00)

#plt.savefig('../results/SI/TPC_data_dists_bmax.pdf', bbox_inches='tight')
```

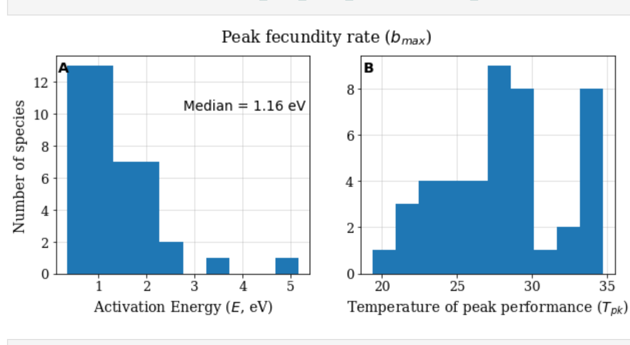

In [37]:
```python
fig = plt.figure(figsize=(10, 4))
fig.suptitle(r'Juvenile mortality rate ($z_J$)', fontsize=16)

ax = fig.add_subplot(121)
ax.set_title('A', weight="bold", fontsize=14, x=.03, y=.9,**csfont)
ax.hist(zj_fits[zj_fits["param"] == "e"]["estimate"])
ax.set_xlabel(r"Activation Energy ($E$, eV)", fontsize=14)
ax.set_ylabel('Number of species', fontsize=14)
ax.tick_params(axis='x', labelsize=13); ax.tick_params(axis='y', labelsize=13)
ax.text(0.5, 0.8, 'Median = {:2.2f} eV'.format(np.median(zj_fits[zj_fits["param"] == "e"]["estimate"])),
        horizontalalignment='left', verticalalignment='top', **csfont,
        fontsize=.8*fntsiz, transform=ax.transAxes)

ax = fig.add_subplot(122)
ax.set_title('B', weight="bold", fontsize=14, x=.03, y=.9,**csfont)
ax.hist(zj_fits[zj_fits["param"] == "topt"]["estimate"])
ax.set_xlabel(r"Temperature of peak performance ($T_{pk}$)", fontsize=14)
# ax.set_ylabel('Number of species', fontsize=14)
ax.tick_params(axis='x', labelsize=13); ax.tick_params(axis='y', labelsize=13)

plt.subplots_adjust(wspace=0.2, hspace=.00)

#plt.savefig('../results/SI/TPC_data_dists_bmax.pdf', bbox_inches='tight')
```

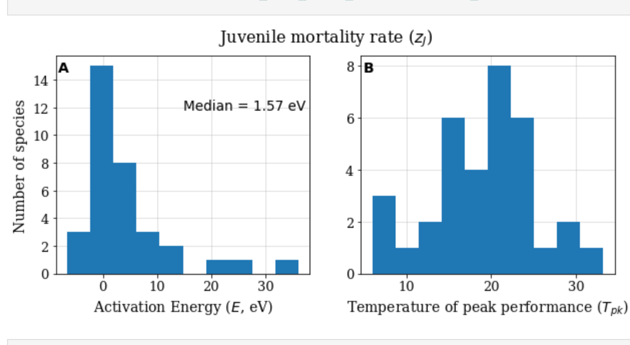

In [38]:
```python
fig = plt.figure(figsize=(10, 4))
fig.suptitle(r'Adult mortality rate ($z$)', fontsize=16)

ax = fig.add_subplot(121)
ax.set_title('A', weight="bold", fontsize=14, x=.03, y=.9,**csfont)
ax.hist(z_fits[z_fits["param"] == "e"]["estimate"])
ax.set_xlabel(r"Activation Energy ($E$, eV)", fontsize=14)
ax.set_ylabel('Number of species', fontsize=14)
ax.tick_params(axis='x', labelsize=13); ax.tick_params(axis='y', labelsize=13)
ax.text(0.5, 0.8, 'Median = {:2.2f} eV'.format(np.median(z_fits[z_fits["param"] == "e"]["estimate"])),
        horizontalalignment='left', verticalalignment='top', **csfont,
        fontsize=.8*fntsiz, transform=ax.transAxes)

ax = fig.add_subplot(122)
ax.set_title('B', weight="bold", fontsize=14, x=.03, y=.9,**csfont)
ax.hist(z_fits[z_fits["param"] == "topt"]["estimate"])
ax.set_xlabel(r"Temperature of peak performance ($T_{pk}$)", fontsize=14)
# ax.set_ylabel('Number of species', fontsize=14)
ax.tick_params(axis='x', labelsize=13); ax.tick_params(axis='y', labelsize=13)

plt.subplots_adjust(wspace=0.2, hspace=.00)

#plt.savefig('../results/SI/TPC_data_dists_bmax.pdf', bbox_inches='tight')
```

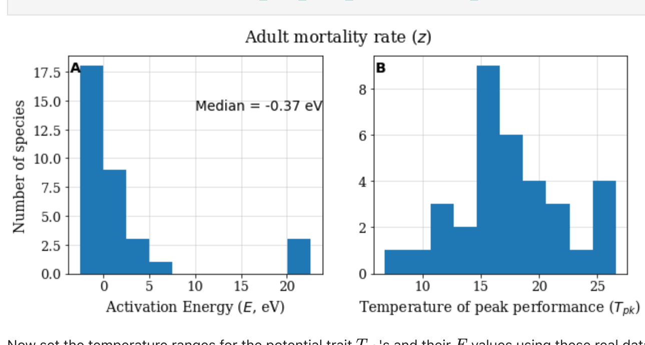

Now set the temperature ranges for the potential trait T_{pk}'s and their E values using these real data:

In [39]:
```python
max_T_pk = 36
T_vec = np.arange(0, max_T_pk, 0.05)+273.15 #Vector of temperatures
kap_vec = B_lam(B_0_kap,0,T_pk_kap+273.15,T_ref_par,E_D_kap,k_par,T_vec) # keep kappa's TPC fixed as it has sma

np.random.seed(720) # Set seed to generate the specific results used in the manuscript (changing the seed does
samps = 50000 # number of simulations
T_pks = np.random.uniform(6, max_T_pk, [samps,4])+273.15 # generate uniform random T_pks in Kelvin for the 4 pa
r_opt_vec = np.zeros([samps, 3])

for i in range(samps): #THIS WILL GENERATE SOME EXTREME VALUE WARNINGS WHEN MORTALITY RATES PEAK AT TOO HIGH A
    alp_tmp_vec = B_inv_lam(B_0_alp,0.7,T_pks[i,0],T_ref_par,E_D_alp,k_par,T_vec)
    bmax_tmp_vec = B_lam(B_0_bmax,1,T_pks[i,1],T_ref_par,E_D_bmax,k_par,T_vec)
    zJ_tmp_vec = B_inv_lam(B_0_zJ,1,T_pks[i,2],T_ref_par,E_D_zJ,k_par,T_vec)
    z_tmp_vec = B_inv_lam(B_0_z,1,T_pks[i,3],T_ref_par,E_D_z,k_par,T_vec)

    r_m_tmp_vec = r_SP_app_lam(bmax_tmp_vec, zJ_tmp_vec, z_tmp_vec, kap_vec, alp_tmp_vec)
    r_opt_vec[i,0] = max(r_m_tmp_vec)
    r_opt_vec[i,1] = T_vec[np.where(r_m_tmp_vec == max(r_m_tmp_vec))]-273.15
    if ((T_pks[i,0] > T_pks[i,1] > T_pks[i,2] > T_pks[i,3])):
        r_opt_vec[i,2] = 1

T_pk_sums = np.sum(T_pks-273.15,1)
T_pk_vars = np.var(np.c_[T_pks-273.15, np.asarray([max_T_pk]*samps) ],1)
```

In [40]:
```python
sim_results = pd.DataFrame(np.hstack((np.transpose(np.vstack((T_pk_sums, T_pk_vars))), r_opt_vec)),
                          columns = ['T_pk_sums','T_pk_vars','r_m_opt','T_opt','Opt_T_pk_order'])

sim_results.head()
```

Out[40]:

	T_pk_sums	T_pk_vars	r_m_opt	T_opt	Opt_T_pk_order
0	87.195357	58.842862	0.076453	15.95	0.0
1	90.660932	44.143652	0.148928	19.80	0.0
2	89.466868	89.880779	0.063248	14.50	0.0
3	68.689012	77.539396	0.012438	11.75	0.0
4	58.033695	108.287276	0.002569	11.00	0.0

In [41]:
```python
sim_results.to_csv("../results/sim_results.csv", index=False)
```

In [42]:
```python
fig = plt.figure(figsize=(10, 4))
ax = fig.add_subplot(121)
ax.set_title('A', weight="bold", fontsize=14, x=.03, y=.9,**csfont)
ax.scatter(sim_results["T_pk_sums"],sim_results["r_m_opt"],
           s=70, alpha=0.75, facecolors='k', edgecolors = 'k')
ax.scatter(sim_results.loc[sim_results['Opt_T_pk_order'] == 1]['T_pk_sums'],
           sim_results.loc[sim_results['Opt_T_pk_order'] == 1]['r_m_opt'],
           color = 'gainsboro', s=70, alpha=1)
ax.set_xlabel(r"Sum of $T_{pk}$'s", fontsize=14)
ax.set_ylabel('Maximum population \n growth rate at $T_{opt}$ ($r_{m,opt}$)', fontsize=14);
ax.tick_params(axis='x', labelsize=13); ax.tick_params(axis='y', labelsize=13)

ax = fig.add_subplot(122)
ax.set_title('B', weight="bold", fontsize=14, x=.03, y=.9,**csfont);
ax.scatter(sim_results["T_pk_vars"],sim_results["r_m_opt"],
           s=70, alpha=0.75, facecolors='k', edgecolors = 'k')
ax.scatter(sim_results.loc[sim_results['Opt_T_pk_order'] == 1]['T_pk_vars'],
           sim_results.loc[sim_results['Opt_T_pk_order'] == 1]['r_m_opt'],
           color = 'gainsboro', s=70, alpha=1)
ax.set_xlabel(r"Variance of $T_{pk}$'s", fontsize=14);
ax.tick_params(axis='x', labelsize=13); ax.tick_params(axis='y', labelsize=13)
ax.axes.yaxis.set_ticklabels([])

plt.subplots_adjust(wspace=0.02, hspace=.00)

# plt.savefig('../results/Figs/TherMM_grad.pdf', bbox_inches='tight')
```

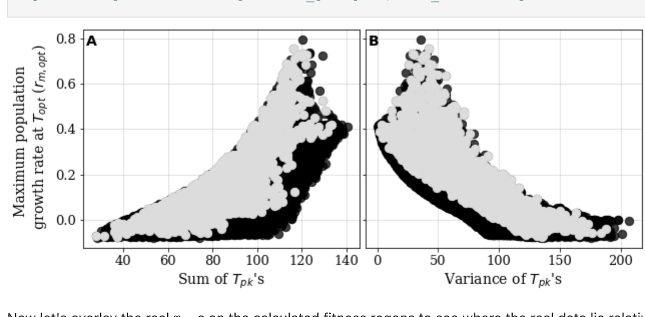

Now let's overlay the real r_{opt}s on the calculated fitness regons to see where the real data lie relative to the predicted fittest strategies.

In [43]:
```python
real_r_m_opt = pd.read_csv('../results/rm_optSizeScaling_w_tpksums.csv')
real_r_m_opt.head()
```

Out[43]:

	species	temp	alpha	alphaLwr	alphaUpr	curve_ID	bmax	bmaxLwr	bmaxUpr	z	...	kappa	rm_
0	Aedes aegypti	33.108108	5.430507	6.139773	4.974288	3	8.966715	6.913063	10.160802	0.047646	...	0.01	0.197
1	Anoplophora glabripennis	27.972973	297.190161	316.589257	275.438861	1	3.279634	2.502936	4.185761	0.018340	...	0.01	0.012
2	Anthonomus grandis	29.459459	11.006110	13.270318	9.919390	4	6.292496	5.369757	10.030917	0.040695	...	0.01	0.143
3	Aphis gossypii	28.243243	4.573511	4.799221	4.398614	6	3.206500	2.552281	3.740294	0.122792	...	0.01	0.235
4	Aphis nasturtii	25.855856	6.736810	7.193387	6.425259	12	2.982606	2.598976	3.421900	0.065587	...	0.01	0.147

5 rows × 25 columns

In [44]:
```python
fig = plt.figure(figsize=(5, 4))
ax = fig.add_subplot()

ax.scatter(sim_results["T_pk_sums"],sim_results["r_m_opt"],
           s=70, alpha=0.75, facecolors='k', edgecolors = 'k')
ax.scatter(sim_results.loc[sim_results['Opt_T_pk_order'] == 1]['T_pk_sums'],
           sim_results.loc[sim_results['Opt_T_pk_order'] == 1]['r_m_opt'],
           color = 'gainsboro', s=70, alpha=1)
ax.scatter(real_r_m_opt["sum"], real_r_m_opt["rm_massCor"],
           color = 'blue', s=100, alpha=.7)

ax.set_xlabel(r"Sum of $T_{pk}$'s", fontsize=14)
ax.set_ylabel('Maximum population \n growth rate at $T_{opt}$ ($r_{m,opt}$)', fontsize=14);
ax.tick_params(axis='x', labelsize=13); ax.tick_params(axis='y', labelsize=13)
plt.savefig('../results/TherMM_grad_with_data.pdf', bbox_inches='tight')
```

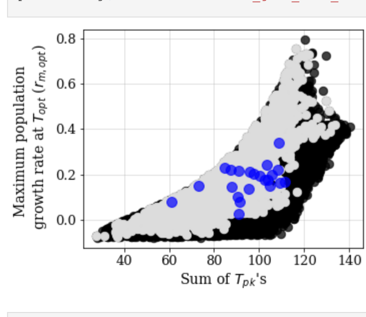

In [45]:
```python
fig = plt.figure(figsize=(5, 4))
ax = fig.add_subplot()

ax.scatter(sim_results["T_pk_vars"],sim_results["r_m_opt"],
           s=70, alpha=0.75, facecolors='k', edgecolors = 'k')
ax.scatter(sim_results.loc[sim_results['Opt_T_pk_order'] == 1]['T_pk_vars'],
           sim_results.loc[sim_results['Opt_T_pk_order'] == 1]['r_m_opt'],
           color = 'gainsboro', s=70, alpha=1)
ax.scatter(real_r_m_opt["variance"], real_r_m_opt["rm_opt"]/(real_r_m_opt["mass"]**-0.158406291367774),
           color = 'blue', s=100, alpha=.7)

ax.set_xlabel(r"Variance of $T_{pk}$'s", fontsize=14)
ax.set_ylabel('Maximum population \n growth rate at $T_{opt}$ ($r_{m,opt}$)', fontsize=14);
ax.tick_params(axis='x', labelsize=13); ax.tick_params(axis='y', labelsize=13)
#plt.savefig('../results/Figs/TherMM_grad_with_data_2.pdf', bbox_inches='tight')
```

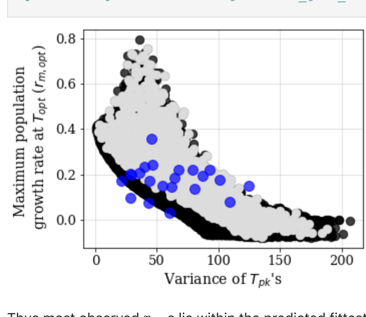

Thus most observed r_{opt}s lie within the predicted fittest straegy regions.

Sensitivity of the results to the parameterisation of fecundity loss rate (κ)

Fecundity typically declines over time, which can have significant impacts on the lifetime reproduction of individuals and therefore fitness. The rate at which fecundity declines with age (κ) may be temperature-dependent, but there appears to be practically no exisiting data on this for arthropods. Therefore, here we quantify the sensitivity of our theoretical predictions to changes in parametrisation of baseline fecundity loss rate (the normalisation constant κ_0). Specifically, we re-evaluate our trait sensitivity analyses, as well as our calculation of selection gradient by varying κ_0 across two extreme values, around the value we have used to generate the main results (0.1).

Here's what changing κ_0 does (qualitatively) to the shape of the fecundity curve at any given temperature:

In [46]:
```python
fig = plt.figure(); ax = fig.add_subplot(111)
b_x_lam = lambdify((b_max, kappa, alpha, x), b_x, np) #use lambidify to speed up

kap_range = [0.0,0.01,0.1,1.0]

ax.plot(x_vec[x_vec>alp_par], b_x_lam(b_max_par, kap_range[0], alp_par, x_vec[x_vec>alp_par]));
ax.text(max(x_vec), 1, r'$\kappa_0 = $'+ str(kap_range[0]), horizontalalignment = "right",
        verticalalignment = "top", fontsize = 13);
ax.plot(x_vec[x_vec>alp_par], b_x_lam(b_max_par, kap_range[1], alp_par, x_vec[x_vec>alp_par]));
ax.text(max(x_vec), .7, r'$\kappa_0 = $'+ str(kap_range[1]), horizontalalignment = "right",
        verticalalignment = "top", fontsize = 13);
ax.plot(x_vec[x_vec>alp_par], b_x_lam(b_max_par, kap_range[2], alp_par, x_vec[x_vec>alp_par]));
ax.text(max(x_vec), 0.05, r'$\kappa_0 = $'+ str(kap_range[2]), horizontalalignment = "right",
        verticalalignment = "bottom", fontsize = 13);
ax.plot(x_vec[x_vec>alp_par], b_x_lam(b_max_par, kap_range[3], alp_par, x_vec[x_vec>alp_par]));
ax.text(30, 0, r'$\kappa_0 = $'+ str(kap_range[3]), horizontalalignment = "left",
        verticalalignment = "bottom", fontsize = 13);
ax.set_xlabel('Age (Days)', fontsize=14);
ax.set_ylim([0,b_max_par*1.1]);
ax.set_ylabel('Fecundity $(b_x)$', fontsize=14)
plt.savefig('../results/SI/kappa_plot.pdf', bbox_inches='tight')
```

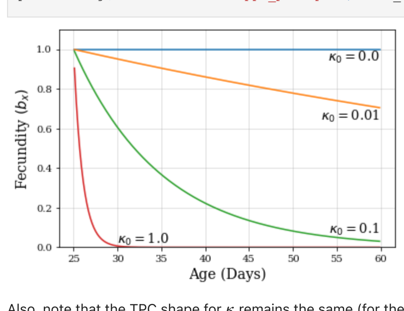

Also, note that the TPC shape for κ remains the same (for the two meangful extreme values of κ_0):

In [47]:
```python
# kap_lo = min(kap_range)
kap_lo = kap_range[1]
kap_hi = max(kap_range)

kap_vec_lo = B_lam(kap_lo,E_kap,T_pk_kap+273.15,T_ref_par,E_D_kap,k_par,T_vec)
kap_vec_hi = B_lam(kap_hi,E_kap,T_pk_kap+273.15,T_ref_par,E_D_kap,k_par,T_vec)

fig = plt.figure(figsize=(8, 4))

ax = fig.add_subplot(121)

ax.plot(T_vec-273.15, kap_vec_lo);
ax.set_xlim([0,35])
ax.set_xlabel(r'Temperature ($^\circ$C)', fontsize=14);
ax.set_ylabel(r'$\kappa$', fontsize=14)
ax.set_title(r'$\kappa_0$ = ' + str(kap_lo), fontsize=13)

ax = fig.add_subplot(122)

ax.plot(T_vec-273.15, kap_vec_hi);
ax.set_xlim([0,35])
ax.set_xlabel(r'Temperature ($^\circ$C)', fontsize=14);
ax.set_title(r'$\kappa_0$ = ' + str(kap_hi), fontsize=13) ;
fig.tight_layout()
plt.savefig('../results/SI/kappa_plot_2.pdf', bbox_inches='tight')
```

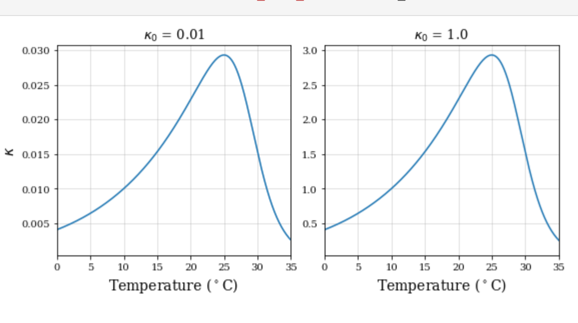

Effect on the trait sensitivity results

First we re-evaluate the trait sensitivity analysis results.

In [48]:
```python
T_vec = 273.15+np.arange(0, 40, 0.05) #Vector of temperatures

alp_vec = B_inv_lam(B_0_alp,E_alp,T_pk_alp+273.15,T_ref_par,E_D_alp,k_par,T_vec)
bmax_vec = B_lam(B_0_bmax,E_bmax,T_pk_bmax+273.15,T_ref_par,E_D_bmax,k_par,T_vec)
z_vec = B_inv_lam(B_0_z,E_z,T_pk_z+273.15,T_ref_par,E_D_z,k_par,T_vec)
zJ_vec = B_inv_lam(B_0_zJ,E_zJ,T_pk_zJ+273.15,T_ref_par,E_D_zJ,k_par,T_vec)
kap_vec_lo = B_lam(kap_lo,E_kap,T_pk_kap+273.15,T_ref_par,E_D_kap,k_par,T_vec)
kap_vec_hi = B_lam(kap_hi,E_kap,T_pk_kap+273.15,T_ref_par,E_D_kap,k_par,T_vec)

dr_dkap_vec_lo = dr_dkap_lam(bmax_vec, alp_vec, z_vec, zJ_vec, kap_vec_lo)
dr_dkap_vec_hi = dr_dkap_lam(bmax_vec, alp_vec, z_vec, zJ_vec, kap_vec_hi)

dkap_dT_vec_lo =  dkap_dT_lam(kap_lo,E_kap,T_pk_kap+273.15,T_ref_par,E_D_kap,k_par,T_vec)
dkap_dT_vec_hi =  dkap_dT_lam(kap_hi,E_kap,T_pk_kap+273.15,T_ref_par,E_D_kap,k_par,T_vec)

dr_dkap_dT_vec_lo = dr_dkap_vec_lo * dkap_dT_vec_lo
dr_dkap_dT_vec_hi = dr_dkap_vec_hi * dkap_dT_vec_lo

dr_dT_vec_lo = dr_dbmax_dT_vec + dr_dalp_dT_vec + dr_dz_dT_vec + dr_dzJ_dT_vec + dr_dkap_dT_vec_lo
dr_dT_A_vec_lo = dr_dbmax_dT_vec + dr_dz_dT_vec + dr_dkap_dT_vec_lo

dr_dT_vec_hi = dr_dbmax_dT_vec + dr_dalp_dT_vec + dr_dz_dT_vec + dr_dzJ_dT_vec + dr_dkap_dT_vec_hi
dr_dT_A_vec_hi = dr_dbmax_dT_vec + dr_dz_dT_vec + dr_dkap_dT_vec_hi
```

In [49]:
```python
fig = plt.figure(figsize=(10, 4))
ax = fig.add_subplot(121)
ax.plot(T_vec-273.15,dr_dT_vec_lo, 'black', linewidth=3, linestyle='-.')
ax.set_title(r'$\kappa_0 = $'+str(kap_lo), fontsize=13)
ax.plot(T_vec-273.15,dr_dalp_dT_vec, 'blue')
ax.plot(T_vec-273.15,dr_dbmax_dT_vec, 'red')
ax.plot(T_vec-273.15,dr_dzJ_dT_vec, 'orange')
ax.plot(T_vec-273.15,dr_dz_dT_vec, 'cyan')
ax.plot(T_vec-273.15,dr_dkap_dT_vec_lo, 'purple')
ax.plot([T_pk_par, T_pk_par], [ax.get_ylim()[0],ax.get_ylim()[1]], color='k', linestyle='--', linewidth=1)
ax.set_xlim(0,35)
ax.set_ylim(-0.025,.02)
ax.set_xlabel('Temperature', fontsize=14);
ax.set_ylabel(r'$\frac{dr_m}{dT}$', fontsize=14);
plt.legend(['Full', r'$\alpha$', '$b_{max}$', '$z_J$', '$z$', r'$\kappa$'], loc='lower left') #Add kappa to leg

ax = fig.add_subplot(122)
ax.plot(T_vec-273.15,dr_dT_vec_hi, 'black', linewidth=3, linestyle='-.')
ax.set_title(r'$\kappa_0 = $'+str(kap_hi), fontsize=13)
ax.plot(T_vec-273.15,dr_dalp_dT_vec, 'blue')
ax.plot(T_vec-273.15,dr_dbmax_dT_vec, 'red')
ax.plot(T_vec-273.15,dr_dzJ_dT_vec, 'orange')
ax.plot(T_vec-273.15,dr_dz_dT_vec, 'cyan')
ax.plot(T_vec-273.15,dr_dkap_dT_vec_hi, 'purple')
ax.plot([T_pk_par, T_pk_par], [ax.get_ylim()[0],ax.get_ylim()[1]], color='k', linestyle='--', linewidth=1)
ax.set_xlim([0,35])
ax.set_ylim([-0.048,0.02])
ax.set_xlabel('Temperature', fontsize=14)

plt.savefig('../results/SI/r_sens_kap.pdf', bbox_inches='tight')
```

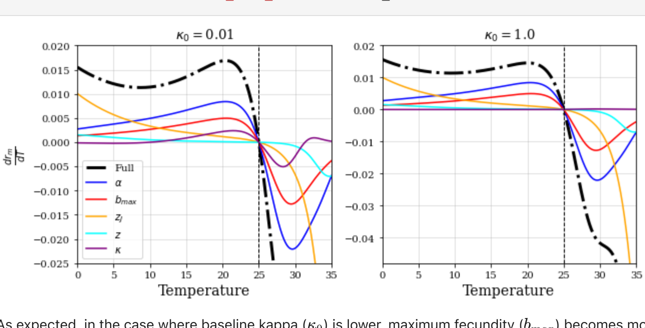

As expected, in the case where baseline kappa (κ_0) is lower, maximum fecundity (b_{max}) becomes more important relative to κ, leaving the order of importance of the 5 traits the same as for the intermediate case ($\kappa_0 = 0.1$) upon which our main results are based.

The selection gradients revisited

Next we re-evaluate the r_m TPC and selection gradient as above. We focus only on the dominant trait α because the order of the strengths of selection gradients is bound to remain unchanged due to the unchanged order of trait sensitivity irrespective of the κ_0 value (previous section).

In [50]:

```
fig = plt.figure(figsize=(9, 8))

ax = fig.add_subplot(221)
ax.set_title(r'$\kappa_0$ = ' + str(kap_lo), fontsize=13)
ax.set_xlabel(r'Temperature ($^\circ$C)', fontsize=14);
ax.set_ylabel('$r_m$', fontsize=14)

r_m_vec = r_SP_app_lam(bmax_vec, zJ_vec, z_vec, kap_vec_lo, alp_vec)

r_opt_vec = ["NA"]*len(T_pk_mm)

colormap = plt.cm.copper_r
ax.set_prop_cycle('color',[colormap(i) for i in np.linspace(0, 1, len(T_pk_mm))])

for i in range(len(T_pk_mm)):
    alp_mm_tmp_vec = B_inv_lam(B_0_alp,E_alp,T_pk_mm[i]+273.15,T_ref_mm,E_D_alp,k_par,T_vec)
    zJ_mm_tmp_vec = B_inv_lam(B_0_zJ,E_zJ,T_pk_mm[i]+273.15,T_ref_mm,E_D_zJ,k_par,T_vec)
    r_mm_tmp_vec = r_SP_app_lam(bmax_vec,zJ_mm_tmp_vec, z_vec, kap_vec_lo, alp_mm_tmp_vec)
    r_opt_vec[i] = max(r_mm_tmp_vec)

    ax.plot(T_vec-273.15, r_mm_tmp_vec)

ax.plot(T_vec-273.15, r_m_vec, 'blue', linestyle='-', linewidth = 3)
ax.plot(T_vec-273.15, [0]*len(T_vec), "k--")
plt.ylim(-0.05,0.135)
plt.xlim(0,40)

ax = fig.add_subplot(223)
ax.plot(T_pk_mm - T_pk_par, r_opt_vec, 'black');
ax.set_xlabel(r'$\Delta T_{pk}^\alpha$ ($^\circ$C)', fontsize=14);
ax.set_ylabel('$r_{m, opt}$', fontsize=14)

ax = fig.add_subplot(222)
ax.set_title(r'$\kappa_0$ = ' + str(kap_hi), fontsize=13)
ax.set_xlabel(r'Temperature ($^\circ$C)', fontsize=14);

r_m_vec = r_SP_app_lam(bmax_vec, zJ_vec, z_vec, kap_vec_hi, alp_vec)

r_opt_vec = ["NA"]*len(T_pk_mm)

ax.set_prop_cycle('color',[colormap(i) for i in np.linspace(0, 1, len(T_pk_mm))])

for i in range(len(T_pk_mm)):

    alp_mm_tmp_vec = B_inv_lam(B_0_alp,E_alp,T_pk_mm[i]+273.15,T_ref_mm,E_D_alp,k_par,T_vec)
    zJ_mm_tmp_vec = B_inv_lam(B_0_zJ,E_zJ,T_pk_mm[i]+273.15,T_ref_mm,E_D_zJ,k_par,T_vec)

    r_mm_tmp_vec = r_SP_app_lam(bmax_vec,zJ_mm_tmp_vec, z_vec, kap_vec_hi, alp_mm_tmp_vec)

    r_opt_vec[i] = max(r_mm_tmp_vec)

    ax.plot(T_vec-273.15, r_mm_tmp_vec)

ax.plot(T_vec-273.15, r_m_vec, 'blue', linestyle='-', linewidth = 3)
ax.plot(T_vec-273.15, [0]*len(T_vec), "k--");
plt.ylim(-0.05,0.135)
plt.xlim(0,40)

ax = fig.add_subplot(224)
ax.plot(T_pk_mm, r_opt_vec, 'black');
ax.set_xlabel(r'$T_{pk}^\alpha$ ($^\circ$C)', fontsize=14);

fig.tight_layout()
plt.savefig('../results/SI/kappa_plot_3.pdf', bbox_inches='tight')
```


Thus the selection gradient remains qualitatively unchanged, with overall r_m lower when κ is high, as expected.
